# Viewing Art in Different Contexts

**DOI:** 10.3389/fpsyg.2020.00569

**Published:** 2020-04-02

**Authors:** Vicente Estrada-Gonzalez, Scott East, Michael Garbutt, Branka Spehar

**Affiliations:** ^1^School of Psychology, University of New South Wales, Sydney, NSW, Australia; ^2^Faculty of Art and Design, University of New South Wales, Sydney, NSW, Australia

**Keywords:** eye movements, museums, art, image statistics, gaze patterns

## Abstract

While aesthetic experiences are not limited to any particular context, their sensorial, cognitive and behavioral properties can be profoundly affected by the circumstances in which they occur. Given the ubiquitous nature of contextual effects in nearly all aspects of behavior, investigations aimed at delineating the context-dependent and context-independent aspects of aesthetic experience and engagement with aesthetic objects in a diverse range of settings are important in empirical aesthetics. Here, we analyze the viewing behavior of visitors (*N* = 19) freely viewing 15 paintings in the 20th-century Australian collection room at the Art Gallery of New South Wales. In particular, we focus on how aspects of viewing behavior including viewing distance in the gallery condition and eye gaze measures such as fixation count, total fixation duration and average fixation duration are affected by the artworks’ physical characteristics including size and image statistics properties such as Fourier amplitude spectrum, fractal dimension and entropy. In addition, the same artworks were viewed in the laboratory, either scaled to fit most of the screen (*N* = 22) or to preserve their relative size as in the museum condition (*N* = 17) to assess the robustness of these relationships across different presentation contexts. We find that the effects of presentation context are modulated by the artworks’ physical characteristics.

## Introduction

It is well-established that context affects aesthetic experience and that investigations in authentic and ecologically-valid settings such as art museums are important in empirical aesthetics (for a review see Pelowski et al., 2017). Since the seminal work by Locher et al. (1999, 2001), studies in empirical aesthetics conducted in naturalistic settings have proliferated, afforded by new methods and techniques that have become available. Furthermore, in recent times the way we engage with art has changed dramatically, owing to the proliferation and accessibility of digital information sources and online art repositories. According to Groys (2016), art institutions nowadays use digital platforms as the main place of information distribution about exhibitions and other art events. Museum websites and image repositories of their collections have become an extension of the museum, thus allowing people from around the globe to have access to images of the artworks. Styliani et al. (2009) argue that technology also provides solutions to contemporary issues, such as art gallery exibition and storage space limitations by creating virtual environments with an infinite exhibition and storage capacity. In fact, museums have not only created repositories of their collections visitors can scroll through when looking at works of art in the traditional way, but virtual exhibitions, where avatars mimic navigation in real museums, have become popular in recent years. For example, museums such as the Louvre, the Guggenheim (NYC), and the British Museum, among others, provide 3D tours of temporary and permanent shows on their websites. The University of Hamburg developed a virtual replica of *Alt-Segeberg Bürgerhaus* museum that enables people to visit the museum by using a virtual reality headset remotely, which aims to provide a more “real” experience that includes body movements in the tour (Kersten et al., 2017). Finally, the Google Art Project (GAP) is an ambitious long-term venture that aims to digitize at the highest possible resolution museum collections from all over the world. In 2011, Beth Harris, the director of Digital Learning at the Museum of Modern Art in New York, argued that the GAP will help make the art experience more pleasant by avoiding “crowds, physical fatigue and self-consciousness” (Proctor, 2011). In summary, it is clear that digital availability of artworks has a substantial presence in the art field, making it important to continue investigating contextual factors in aesthetic experience and engagement with artworks in contexts ranging from museums and laboratories to tablets and cell phones.

Pelowski et al. (2017) comprehensive review of studies comparing the experience of museum-based art to that of digital reproductions in the laboratory noted a number of important contextual effects. For example, artworks are rated as more “immediate” and “pleasant” when viewed in museums (Locher et al., 1999) and, conversely, viewers perceive artworks displayed on computer screens as less interesting, less arousing, more ambiguous and less memorable than the same works exhibited in the gallery (Brieber et al., 2014, 2015). Different presentation contexts have also been reported to result in different viewing behaviors. Empirical studies utilizing mobile eye-tracking have reported longer viewing times (Brieber et al., 2014; Balbi et al., 2016) as well as more widespread distribution of fixations (Quiroga et al., 2011; Walker et al., 2017) for artworks viewed in the museum context compared to the digital reproduction in laboratory.

However, the differences in art experience in different contexts are not always as pronounced. For example, Locher et al. (1999) found significant differences across different contexts evident in only four of the 16 rating scales (sparse-dense, distant-immediate, similar-contrasting, and unpleasant pleasant). The evaluations related to physical, structural and compositional characteristics of artworks were virtually indistinguishable across presentation formats. In subsequent studies, Locher et al. (2001) and Locher and Dolese (2004) found that the ratings of symmetry, heterogeneity, randomness, complexity and clutter were very similar across the original and different reproduction formats (ranging from slide projections to postcards) and did not differ between naïve and more sophisticated viewers. Based on these results, Locher et al. (1999) proposed the notion of “pictorial sameness” and argued that under some conditions, the reproduction can be as perceptually valuable as the original, with the viewers exhibiting “facsimile accommodation” and the ability to “look beyond” the limitations of the medium.

The facsimile accommodation hypothesis notwithstanding, Locher et al. (1999, 2001) were careful to emphasize that the reproduction of a painting is not the same as the original, and that the authentic art context certainly has the potential to enhance the art appreciation. Though extremely plausible, this assertion was tested by Brieber et al. (2015) who noted that in most of the studies of contextual effects on art experience, the effects of genuineness (authenticity or originality) and context were confounded in that participants always view the genuine artworks in museums and the reproductions in the laboratory. In an attempt to dissociate the effect of genuineness from the physical context they tested liking, interest, arousal, valence and understanding of both genuine and reproduced artworks in both gallery and laboratory (Brieber et al., 2015). Surprisingly, they found that neither physical context, nor genuineness had an effect on participants’ evaluations of artworks and argued that the inconsistencies across studies could be related to the differences in the nature of materials used across different studies (i.e., photographs vs. conceptual installations; thematic focus of the entire exhibition etc.) and personal relevance of the work to the observer (not always high or meaningful to often used psychology students).

### What Causes Difference in Art Experience and Viewing Patterns in Different Contexts?

The many reported differences in the experience and engagement with art between the museum and laboratory contexts play an important role in recent claims regarding the gap between empirical aesthetic science and aesthetic experience (Makin, 2017). Given that most aesthetic research is still conducted in laboratories without access to real artworks, overlooking the contribution of context in which art is typically experienced and appreciated, it is becoming increasingly important to be able to identify the most influential factors associated with different contexts.

Of course, this is not an easy task, since a myriad of particular characteristics define different contexts and differences between them. In a comprehensive review of the characteristics of museum experience, Pelowski et al. (2017) identify three broad groups: (1) features of the artwork; (2) characteristics of the viewer; and (3) characteristics of the presentation context. Features of artworks comprise both physical (size, texture, physical presence, and remnants of the artist’s touch and effort) and perceived features (seeing objects as “art” and perceived authenticity). Characteristics of the viewer include personal characteristics such as age, wealth, art expertise, motivations and expectations and group characteristics such as group size and between group differences. Finally, characteristics of the presentation context include physical and cultural aspects of the museum, display/hanging, frame, lighting, art labels, furniture, movement, viewing distance, viewing time and museum fatigue. While all of these factors might be contributing to differences between art experience in museum and other contexts, to date, the majority of them have remained underexplored (Bitgood et al., 2013; Minissale, 2013; Tröndle et al., 2014; Carbon, 2017).

In addition, the majority of studies to date have compared museum and laboratory contexts by aggregating the measures of art experience and viewing behavior across all artworks under consideration, even though the artworks may vary in a number of important physical characteristics, without providing a finer-grained analysis of the role these characteristics play in both the museum and laboratory contexts.

## The Present Study

The museum component of this study was conducted in a room containing 20th-century Australian artworks at the Art Gallery of New South Wales (AGNSW) in Sydney, Australia. The two important issues that we aim to explore in this study are the effect of the context on viewing behavior (i.e., museum vs. laboratory) and the influence of characteristics of the artwork on viewing behavior in both contexts.

While studies considering the experience of artworks in different contexts often utilize the explicit ratings of artworks to directly measure various aspects of aesthetic experience, we opted to focus on the viewing behavior as an index of spontaneous engagement with such objects. Preferential looking at artworks in an exhibition space is not only a defining feature of an art museum visit but, arguably, can be taken as an immediate and objective index of our engagement with such objects. This idea is not only central to the preferential looking paradigm in general, but has also received support in the aesthetics domain (Holmes and Zanker, 2012; Brieber et al., 2014).

The fine-grained analysis of precisely what and how participants look at art exhibits still remains a topic of enormous theoretical and practical interest, especially for visitor-centered art institutions such as art museums and galleries. While it has been well-established that allocation of attention in any physical context is a complex interplay between “top down” (viewer-centered) and “bottom up” (stimulus-driven) factors, our approach is aligned with the attempts to explore the role of physical, statistical properties in the perception of and interaction with images, including artworks. The physical characteristics of the artworks considered were physical size, and image statistical properties such as Fourier amplitude spectrum, fractal dimension and entropy. All these are general physical characteristics of objects and images known to be effective in capturing attention (Berlyne, 1971; Treisman and Gelade, 1980), or influencing perceived complexity, predictability and/or aesthetic appeal (Redies, 2007; Spehar and Taylor, 2013; Mather, 2014, 2018; Redies, 2015; Viengkham and Spehar, 2018) but have seldom been investigated in studies considering different presentation contexts and viewing behavior (eye movements).

The present study explores the viewing behavior of gallery visitors freely viewing paintings with a particular focus on how the aspects of viewing behavior, including viewing distance and eye gaze measures such as fixation count, total fixation duration and average fixation duration are affected by the artworks’ physical characteristics including physical size and image statistics properties.

### Physical Size

While the studies of visitor behavior in museums have acknowledged that larger artworks are generally more effective in attracting and holding attention (Bitgood, 1993; Bitgood et al., 2013), relatively few studies have systematically investigated the effect of physical size on aesthetic evaluation. One of the rare exceptions is a recent study by Seidel and Prinz (2018), who found that merely altering physical scale of a painting (small vs. large) influenced aesthetic judgment. Participants evaluated larger reproductions more postively, regardless of whether the painting was high in complexity (Picasso’s *Three Musicians*) or low (Joan Miro’s *Blue II*).

The physical size of artworks has also been found to affect viewing distance. Clarke et al. (1984) varied the size of projected art images and asked their participants to choose the distance from which either the artworks “look best” or felt the most comfortable. While there was considerable variability in the preferred viewing distance, all participants chose to view the larger artworks from a greater distance, regardless of instruction. Moreover, Clarke et al. also found that viewing time increased with the projection size but there was no effect of either stimulus size or viewing distance on ratings of how pleasant or interesting the artwork appeared. More recently, in a real art gallery setting, Carbon (2017) confirmed a high positive correlation between the artwork size and viewing distance: the larger the artwork, the greater the viewing distance observed.

### Image Statistics

Despite the apparent heterogeneity and even randomness, artworks, like natural scenes, have characteristic, and regular structure related to the degree of spatial redundancy they exhibit. The spatial redundancy is related to the extent to which the surface properties at any locations can be predicted by the known values at nearby locations, and is intimately coupled with the notions of both spatial information and the scale-invariant, fractal-like properties of both artworks and natural scenes (Redies, 2007; Graham and Field, 2008; Mather, 2018). Here we use three widely-known indices of spatial redundancy: the Fourier spatial frequency amplitude spectrum (1/f^a^), fractal dimension (FD), and Shannon Entropy (SE).

### The Fourier Amplitude Spectrum

The Fourier amplitude spectrum measures the relative contribution of different spatial frequencies in an image as whole. In particular, the slope “alpha” of the 1/f^a^ amplitude spectrum quantifies contribution of coarse spatial structure (low spatial frequency) vs. fine spatial detail (high spatial frequency) in an image and has a value of approximately 1 for both natural scenes and artworks (Graham and Redies, 2010). This particular property of natural scenes and artworks is taken to reflect the scale-invariance of natural scenes, or the notion that approximately equivalent amount of spatial structure exists at different spatial scales. Images with high values of a contain a higher degree of similarity in luminance intensity across image regions and thus a higher degree of spatial redundancy and predictability of intensity variations across an image. Conversely, images with low values of a are associated with a higher degree of intensity variations and thus lower predictability of intensity variations across an image.

### Fractal Dimension

The scale invariance of spatial patterns can also be expressed by a geometric scaling parameter known as the fractal dimension (FD) which can be used to describe and quantify patterns which exhibit self-similarity in geometrical-spatial structure at different levels of magnification (Mandelbrot, 1967). Fractal dimension (FD) measures the degree to which a pattern is broken up (or fractured) into a finer and finer spatial structure. Images containing coarse spatial structures with lack of fine spatial detail are associated with low FD values, whereas images with high levels of intricate and fine spatial detail would have high FD values. FD is inversely related to the slope a of the Fourier amplitude spectrum (higher a values are equivalent to low FD values and vice versa) and the relationship between them has been both established mathematically and validated empirically (Graham and Field, 2008; Forsythe et al., 2011; Spehar and Taylor, 2013; Bies et al., 2016).

### Shannon Entropy (SE)

Shannon entropy (SE) measures the degree to which an image or a spatial form vary unpredictably, or randomly and is inversely related to the notion of spatial redundancy (Mather, 2018). Those images which vary highly unpredictably (or randomly) have a high SE value (or low redundancy), or conversely, images with similar intensity values across the spatial extent would have a low SE value (or high redundancy).

Our selection of these measures of statistical structure was motivated as follows. Firstly, these measures have been used to investigate and characterize the spatial structure of a wide range of different artworks with findings indicating a remarkable similarity in Fourier-based image statistics of artworks from different regions or time periods (Graham and Field, 2007, 2008; Redies, 2007; Graham and Redies, 2010). Most recently, a longitudinal statistical study by Mather (2018) showed that FD and SE remained relatively stable over a period of 500 years, from the 14th–19th century, with marked variations coinciding with the beginning of the Modern Art movement.

More importantly, all three measures exemplify the objective measures of complexity, a notion that belongs amongst the most influential in empirical aesthetics: from Fechner (1876) concept of the aesthetic middle to the Birkhoff (1933) definition of beauty as the ratio of an object’s order (simplicity) and its complexity, and Berlyne (1971) modeling of the relationship between complexity and preference as an inverted U-shape. Our own work and that of others has established that variations in fractal dimension and/or Fourier amplitude spectrum characteristics are highly correlated with the perceived complexity of both synthetic and art images as well as preference for those images (Forsythe et al., 2011; Spehar et al., 2016; Viengkham and Spehar, 2018). However, there has been a relative paucity of investigations into the influence of image statistics properties on engagement with artworks in museum settings.

### Painting Style

**FIGURE 1:**
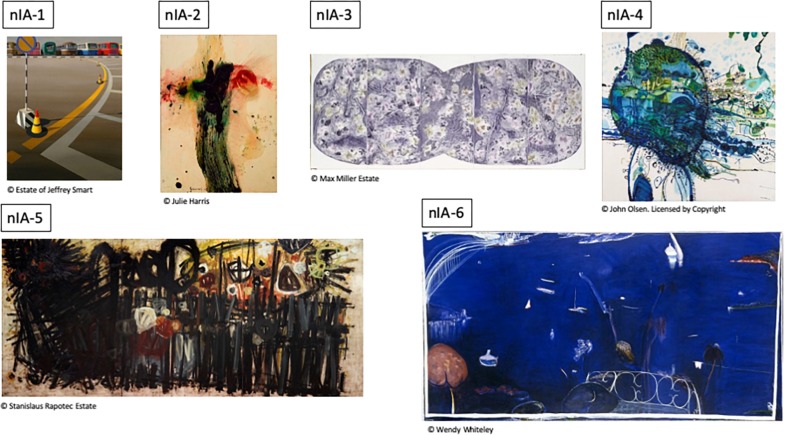
Non-Indigenous Australian paintings used in this study. For detailed description of artworks see Table 1.

**FIGURE 2:**
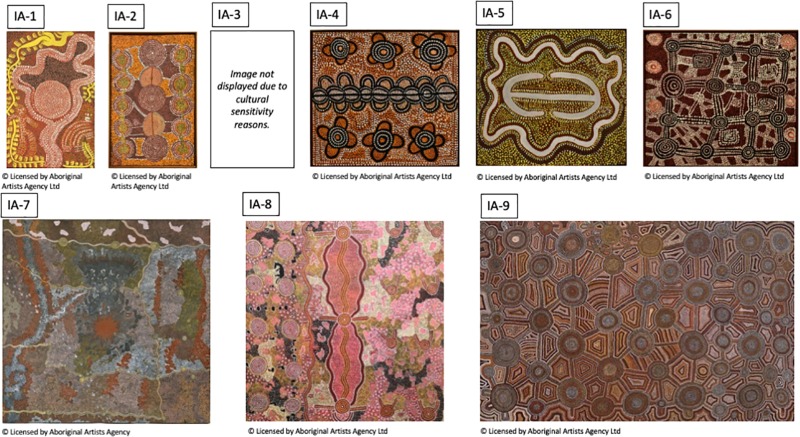
Indigenous Australian paintings used in this study. For detailed description of artworks, see Table 2.

Artworks are often analyzed or classified as belonging to a particular style, typically based on a period, country, cultural group, or art movement. In addition to these predominantly art historical considerations, different art movements are often associated with distinctive visual qualities, which in turn can be associated with their physical features and statistical properties (Mather, 2018). Our study location, a single room in the 20th-century Australian Art section of the Art Gallery of New South Wales in Sydney afforded the opportunity to consider painting styles, non-Indigenous and Indigenous Australian, as an additional characteristic of interest to our study. However, we want to emphasize that these groupings are based on the available sample of artworks, and do not intend to suggest that either group is homogeneous in style or symbolism. For example the Indigenous grouping includes famous works from the Western and Central desert regions and styles and non-Indigenous includes figurative and abstract works. Figures 1, 2 show the artworks belonging to the non-Indigenous and Indigenous Australian artworks, respectively, with the details of these paintings displayed in Table 1 (non-Indigenous artworks) and Table 2 (Indigenous artworks).

**TABLE 1:**
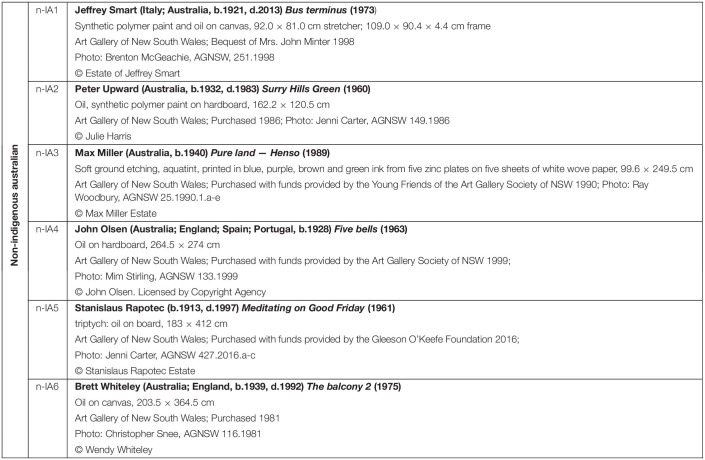
Non-Indigenous paintings.

**TABLE 2:**
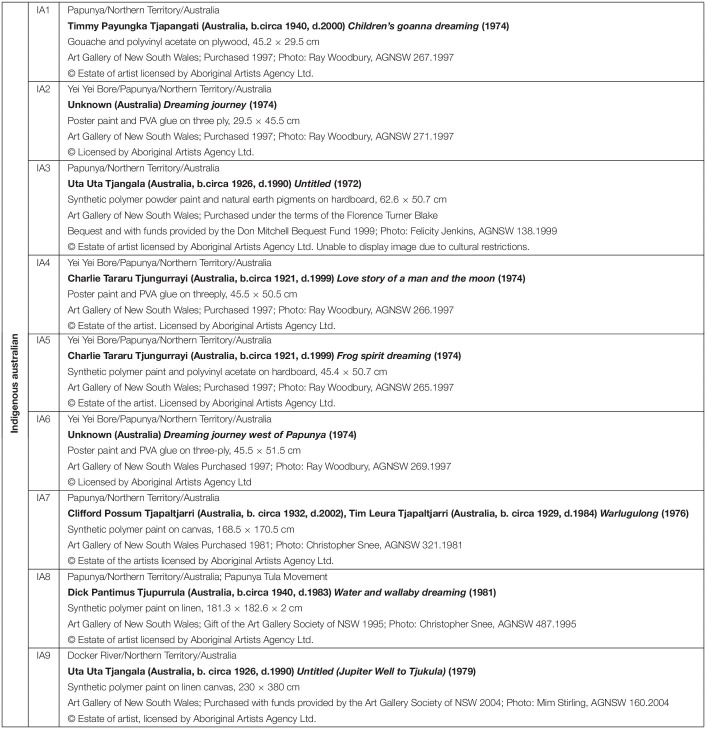
Indigenous paintings.

### Viewing Contexts

In addition to the AGNSW museum condition, the same artworks were viewed on computer monitors in our laboratory, either scaled to preserve their relative size or presented to fit most of the screen in order to assess the robustness of the effect of artworks’ physical characteristics across different presentation contexts. The details of physical dimensions of both artworks and their digital reproductions are shown in the Method section.

Across the three presentation conditions (museum; on-screen relative size; and on-screen full size) we compare the average number of fixations (NF), average total fixation duration (TFD), and average fixation duration (AFD) with mixed effects ANOVA with the presentation condition as the between-subject and Paintings as the within-subject factor. In each presentation condition we also perform regression analyses with the average NF, TFD and AFD as dependent variables and the artwork physical size, Fourier amplitude spectrum and fractal dimension as predictors.

## Method

### Participants

#### In-Museum Condition

A total of nineteen AGNSW visitors (11 female) volunteered to participate in this study. All participants reported normal or corrected-to-normal vision. All participants were over 18. Eleven participants were Sydney residents, two were interstate visitors and six participants were international visitors. Sixteen out of 19 participants reported a university degree or postgraduate studies as their highest level of education. Participant recruitment and all other procedures for the in-museum study were approved by the University of New South Wales, Human Research Ethics Advisory Panel B- Arts, Humanities and Law (Approval No. HC180466).

#### On-Screen Laboratory Conditions

Thirty-nine UNSW students (25 female) volunteered to participate through the UNSW online SONA system in exchange for course credit or a small monetary reward of AU$5). All subjects reported normal or corrected-to-normal vision and were over the age of 18. Participants were randomly assigned to either on-screen full size (22) or on-screen relative size (17) condition. Participant recruitment and all other procedures were approved by the University of New South Wales, Human Research Ethics Advisory Panel C- Psychology (Approval No. 3052).

The sample size in the two viewing conditions is quite small due to both convenience sampling and time limited chance to collect data. Nevertheless, in the museum condition the number of participants in our study is either comparable to or higher than in other eye tracking studies (Quiroga et al., 2011; Brieber et al., 2014; Walker et al., 2017). As such, we do recommend viewing the current findings as exploratory.

### Materials and Stimuli

#### Study Location

**FIGURE 3:**
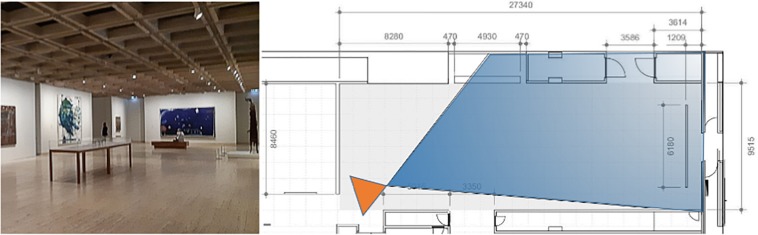
The 20th Century Australian Art room at the Art Gallery of New South Wales: **(top)** photograph; plan indicating the field of the view of the photograph. Adapted from a plan supplied by AGNSW.

The Australian Art gallery at the Art Gallery of New South Wales measures 9.5 × 27.3 m room and contains 15 paintings, as shown in Figure 3.

#### Paintings

The physical dimensions and image statistics of artworks are shown in Table 3.

**TABLE 3:** Physical dimensions and image statistic properties of artworks used in this study.

	ID	Size (cm)	Amplitude spectrum (∝)	Fractal dimension	Entropy	Screen -equal size dimensions (cm) visual angle	Screen -relative size dimensions (cm) visual angle
Non-indigenous australian	NIA1	109 × 90.4	1.7	1.17	6.77	*31.2*× *25.9*	*15.4*× *12.8*
						27.1 × 22.7	13.6 × 11.3
	NIA2	162.2 × 120.5	1.37	1.28	7.32	*31.2*× *23.2*	19.1 × 14.2
						27.1 × 20.4	16.8 × 12.5
	NIA3	106.3 × 253.8	1.2	1.45	7.26	23.5 × 55.4	12.5 × 29.6
						20.5 × 46.4	11.1 × 25.8
	NIA4	265.74 × 274	1.14	1.36	7.64	31.2 × 32.1	31.2 × 32.1
						27.1 × 27.9	27.1 × 27.9
	NIA5	183 × 412	1.28	1.33	7.15	24.4 × 55.4	21.6 × 49
						21.4 × 46.4	18.9 × 41.5
	NIA6	210.9 × 372.2	1.13	1.39	5.63	31 × 54.7	24.9 × 43.9
						26.9 × 45.8	21.7 × 37.5
Indigenous australian	IA1	45.2 × 29.5	0.89	1.65	7.47	31.2 × 20.4	10.7 × 7.0
						27.1 × 17.9	9.4 × 6.2
	IA2	45.5 × 29.5	0.98	1.44	7.2	31.2 × 20.2	10.7 × 6.9
						27.1 × 17.8	9.5 × 6.2
	IA3	62.1 × 36.8	1.1	1.43	7.3	31.2 × 18.5	14.6 × 8.7
						27.1 × 16.3	12.9 × 7.7
	IA4	45.4 × 50.5	0.97	1.49	7.32	31.2 × 34.7	10.7 × 11.9
						27.1 × 30.1	9.5 × 10.5
	IA5	45.5 × 50.5	1.08	1.43	7.1	31.2 × 34.6	10.7 × 11.9
						27.1 × 30.0	9.4 × 10.6
	IA6	45.5 × 51.5	0.91	1.38	7.19	31.2 × 35.5	10.7 × 12.2
						27.1 × 30.7	9.5 × 10.8
	IA7	168.5 × 170.5	0.85	1.73	7.17	31.2 × 31.6	19.9 × 20.1
						27.1 × 27.4	17.4 × 17.7
	IA8	181.3 × 182.6	0.91	1.69	7.74	31.2 × 31.4	21.4 × 21.6
						27.1 × 27.3	18.7 × 19.0
	IA9	230.0 × 380.0	0.9	1.58	7.25	31.2 × 51.5	27.1 × 44.8
						27.1 × 43.3	23.6 × 38.2

#### Image Statistics

We note a high degree of variability in physical size and image statistic measures between individual paintings, both within and between the two painting style groups. The average image statistic values for the two groups are detailed and compared in Table 4. The difference in the average painting area between the non-Indigenous and Indigenous painting style groups was not statistically significant (*t*_13_ = 1.849, *p* = 0.087), nor was the difference in Shannon Entropy (*t*_13_ = −1.395, *p*-0.187). The two groups differed with respect to their average Fourier slope (*t*_13_ = 4.435, *p* < 0.001) and fractal dimension (*t*_13_ = −3.301, *p*-0.006) values. In particular, the Indigenous paintings have a lower average Fourier amplitude spectrum slope and higher average fractal dimension value, consistent with the high level of fine spatial detail (dots) in these artworks.

**TABLE 4:** Average image characteristics of Indigenous and non-Indigenous paintings used in this study.

		Non-indigenous paintings	Indigenous paintings	Student’s *t*-test
Area (m^2^)	Mean	4.71	1.79	*t*_13_ = 1.849, *p* = 0.087
	Median	4.97	0.23	
	*SD*	3.16	2.89	
Fourier slope (α)	Mean	1.303	0.954	*t*_13_ = 4.435, *p* < 0.001
	Median	1.240	0.910	
	*SD*	0.214	0.087	
Fractal dimension (D)	Mean	1.330	1.536	*t*_13_ = −3.301, *p* = 0.006
	Median	1.345	1.490	
	*SD*	0.097	0.130	
Shannon entropy (SE)	Mean	6.962	7.304	*t*_13_ = −1.395, *p* = 0.187
	Median	7.205	7.250	
	*SD*	0.710	0.195	

**FIGURE 4:**
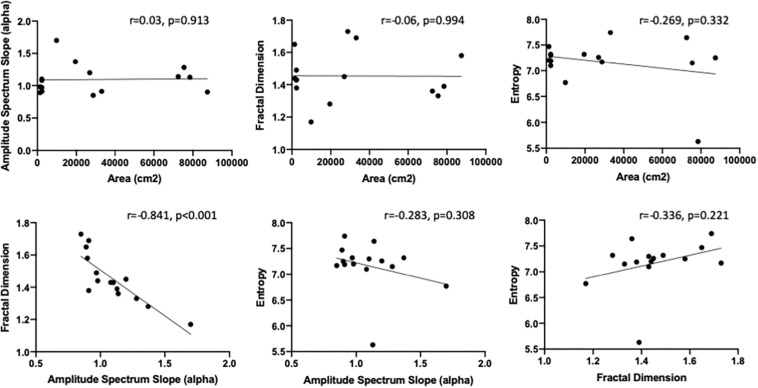
Scatterplots between different image statistics for the 15 paintings used in this study: **(top row)** Painting area vs. Amplitude spectrum slope **(left panel)**, Fractal dimension **(middle panel)** and Shannon entropy **(right panel)**; **(bottom row)** Amplitude spectrum slope vs. fractal dimension **(left panel)**, and Shannon entropy **(middle panel)**, Fractal dimension vs. Shannon entropy **(right panel)**.

In Figure 4, we show the scatterplots between different image statistics measures for all paintings which show that the only significant negative correlation existed between the amplitude spectrum slope (a) and fractal dimension values (*r* = −0.841, *p* < 0.001). Importantly, there were no significant correlations between the painting area and image statistic measures.

#### Physical Size of Artworks in Different Viewing Contexts

Given the very large differences in the area of individual paintings [the area of the largest painting (IA9: 8.74 m^2^) was approximately 65.5 times larger than the area of the smallest painting (IA1 = 0.13 m^2^)], keeping the relative sizes in the on-screen condition true to the physical sizes would have been impossible as the smallest paintings would be virtually impossible to resolve.

The determinations of the relative size were constrained by the screen dimensions which were 55.7 cm × 31.3 cm or 46.4 deg × 27.1 deg of visual angle (VA), viewed at a distance of 65 cm. In order to ensure the relative visibility of the smallest painting, we have limited its size to 10.7 cm × 7 cm corresponding to the visual angle of 9.5 deg × 6.2 deg VA. The biggest paintings occupied most of the full-screen area and the remaining paintings were scaled relative to these two anchors. This ratio was approximately 17:1 in the relative size on-screen condition and 3:1 in the full-size on-screen condition.

**FIGURE 5:**
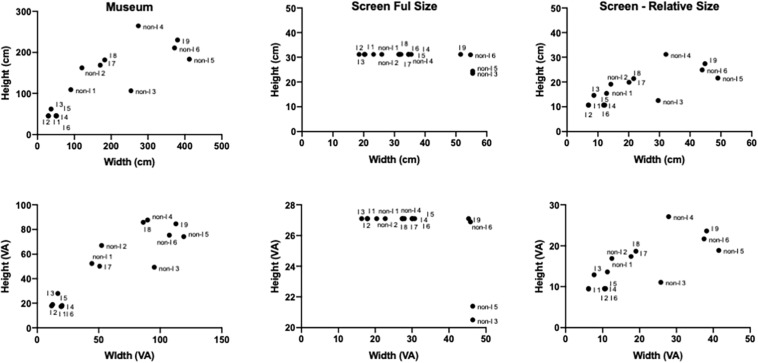
Scatterplots of width and height dimensions in cm **(top row)** and degrees of visual angle **(bottom row)** of paintings in all three conditions. The visual angles in the museum condition were calculated based on the average mean distance estimates for every painting.

We have provided these dimensions in both cm and deg or visual angle for each painting in Table 3. We have also provided scatterplots of each painting’s width and height dimensions in all three conditions in Figure 5, top row. The bottom row plots these values in degrees of visual angle in all three viewing contexts. The visual angles for the museum condition were estimated based on the average mean distance measured for every painting.

### Eye-Tracking Hardware and Software

#### In Museum Condition

A Tobii Pro Glasses 2 mobile eye-tracking system was used to record eye gaze while participants freely explored the gallery room. Gaze behavior was sampled with a frequency of 100 Hz by four cameras (two for each eye). The viewed scene was captured with an extra camera with a visual angle of 82°× 52°. The recorded data were captured and stored via the Tobii Pro Glasses Controller software. For the detailed data analysis, the data were exported to the Tobii Pro Lab Analyser software with integrated Real-World Mapping tool, which scans scene camera video recordings to identify defined areas of interest (AOI) from different perspective angles. Consequently, all fixations allocated in the AOI are aggregated allowing us to extract the following metrics: Number of Fixations (NF), Total Fixation Duration (TFD) and Average Fixation Duration (AFD).

With the Tobii Pro Glasses 2 system eye position (x, y) and gaze vectors (z) are calculated from the eye images using a 3D eye model that gives positions and angles in a coordinate system with its origin in the center of the scene camera. The gaze point is calculated as the vergence point between the two gaze vectors for the left and the right eye. The vergence point indicates how far away the user is looking: the error in estimated distance is small at short distances and increases with distance. We are aware that this is a crude estimate of the distance and in our calculations have eliminated z-values greater than 7 m. In addition, for each participant and each painting we calculated different measures of central tendency (mean, mode, and median) in order to be able to rely on a multiple types of viewing distance estimates. We are aware that these estimates are affected by a number of extraneous factors (head orientation, height, etc.) but still considered it useful to use these estimates as a pilot set of measurements.

#### Laboratory On-Screen Conditions

In the two on-screen conditions a Tobii TX300 desk top system was used to record eye gaze in the two on-screen conditions. The stimulus presentation and data analysis were done with the Tobii Studio software to extract the same metrics as was done in the in-museum condition.

### Procedure

#### In Museum

AGNSW visitors were recruited in the foyer of the museum. After providing informed consent, participants were fitted with the Tobii Pro Glasses 2 at a research table in the same location. A calibration procedure was performed in order to ensure accuracy of eye movements recording. Participants were then invited to “visit that room [*pointing out the 20th^–^ century Australian Art room, the entrance to which was visible about 25 metres away*] and look at the paintings as you would normally do. Take as long as you wish. When you finish, please come back to us.” In order to preserve the museum condition as natural as possible, the experimenter was not present in the exhibition room. For the same reason, there were no attempts to control the crowd density in the exhibition room.

#### Laboratory On-Screen Conditions

The laboratory conditions were conducted in the School of Psychology at UNSW. All participants in the laboratory on-screen condition volunteered to participate through the UNSW SONA system in exchange for course credit, or a small monetary reward ($5). All participants read the subject information sheet and signed the informed consent form before starting the experimental session. They were seated with their chins placed on a rest to avoid abrupt head movements. The chin-rest was positioned 65 cm in front of the screen. The experimental session started with a 9-point target calibration procedure followed by the two practice art images (the *Mona Lisa* and Van Gogh’s *Starry Night*) to experience the self-paced nature of the experiment. Later, instructions appeared on the screen which informed participants that they would be shown a series of 15 paintings which they could view at their own pace without any time constraints and that they should press the spacebar to go to the next painting. The digital reproductions of the 15 paintings from the museum condition were then presented in random order.

## Results

### Museum Condition

#### Total Visit Duration

For the in-museum condition we firstly determined the Room Visit Duration (RVD), defined as the period of time between crossing the threshold to enter the room and crossing it again to leave. Five out of 19 participants left the exhibition room to visit other rooms and then returned to it. Multiple visits were included in calculation of total visit duration. The shortest visit lasted 120 s, the longest 1284 s (2 and 21.4 min). On average, participants spent 521 s (8.68 min) in the room (*SD* = 257 s), with a median of 412 s (6.87 min). On average, 84.5% of the total visit duration was spent viewing paintings (48.7%) and reading labels (35.8%). Participants in our sample also spent, on average, 4.8 % of their total visit time looking at their mobile phones and 2% of the total visit time looking at other people.

#### Number of Visited Paintings

Eleven out of 19 participants (57.9%) looked at all 15 paintings, with four participants (21.1%) and three participants (10.5%) looking at fourteen and thirteen paintings, respectively. One participant (5%) only looked at ten paintings. On average, there was no difference in the average proportion of participants who viewed the paintings between the two painting style groups: the Indigenous Australian paintings were viewed on average by 94% of participants (*SD* = 7%) while the non-Indigenous Australian paintings were viewed on average by 95% of participants (*SD* = 5%).

Across all paintings there was a significant correlation between the average proportion of participants who viewed the painting and the painting physical size (*r* = 0.568, *p* = 0.0271, 95% CI 0.079–0.837) but this relationship was more pronounced for the contemporary non-Indigenous (*r* = 0.808, *p* = 0.051, 95% CI −0.0106–0.978) than for the indigenous paintings (*r* = 0.537, *p* = 0.136, 95% CI −0.197–0.885). There were no significant correlations between the average proportion of participants who viewed the paintings and their image statistics measures.

#### Viewing Distance

For each painting, the viewing distance was estimated by tracking the combined z-coordinate of gaze position for each fixation in the scene camera coordinate system. These fixation-based gaze positions were aggregated for all paintings and all participants with the mean, median and mode extracted as the complementary measures of central tendency for further analysis.

The average mean viewing distance across all paintings was 1.37 m (*SD* = 0.195), ranging from 0.97 to 1.8 m. The average median viewing distance across all paintings was 1.09 m (*SD* = 0.199), ranging from 0.71 to 1.57 m. Finally, the average mode distance equaled 1.03 m (*SD* = 0.234), ranging from 0.77 to 1.43 m. When the results were split according to the painting style, both the average mean, and median viewing distances for the non-Indigenous paintings were shorter than those for the Indigenous paintings. The mean and median viewing distances for the non-Indigenous paintings were 1.241 and 0.957 m, respectively, while the corresponding distances for the Indigenous paintings were 1.4 and 1.178 m, respectively. The paired samples *t*-test revealed a statistically significant difference in the average median distance between the two conditions (*t*_18_ = −2.276, *p* = 0.035, Cohen’s *d* = −0.522), while the difference between the average mean distances did not reach statistical significance (*t*_18_ = 1.907, *p* = 0.07, Cohen’s *d* = −0.437).

**FIGURE 6:**
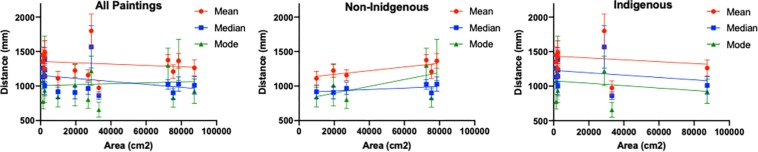
The average mean, median and mode viewing distance as a function of painting area. The error bars represent SEM.

Figure 6 shows the average mean, median and mode distances for every painting plotted as a function of the painting area for all paintings together (left panel) and separately for the two painting styles, the non-Indigenous (middle panel) and Indigenous (right panel). For each painting, the mean, median and mode viewing distances were estimated for every observer. These three individual estimates of central tendency were averaged for each painting and the means with the standard error of the means are shown in Figure 6. When all paintings are considered together, the correlations between the painting area and mean, median and mode viewing distances are non-significant (*r*_mean distance_ = −0.199, *p* = 0.477; *r*_median distance_ = −0.351, *p* = 0.200 and *r*_mode distance_ = 0.092, *p* = 0.742). When the non-Indigenous paintings are considered separately, the pairwise correlations all become positive but fail to reach significance due to low power (*r*_mean distance_ = 0.783, *p* = 0.066; *r*_median distance_ = 0.553, *p* = 0.255 and *r*_mode distance_ = 0.606, *p* = 0.204). With the Indigenous paintings, the correlations between the painting area and mean, median and mode distances become negative, though also non-significant (*r*_mean distance_ = −0.282, *p* = 0.463; *r*_median distance_ = −0.276, *p* = 0.472 and *r*_mode distance_ = −0.268, *p* = 0.485).

The two image statistics measures correlated significantly with the viewing distance: Shannon Entropy was negatively correlated with all measures of viewing distance with Indigenous paintings (*r*_mean distance_ = −0.798, *p* = 0.010; *r*_median distance_ = −0.738, *p* = 0.023 and *r*_mode distance_ = −0.691, *p* = 0.039). In addition, across all paintings, the amplitude spectrum slope was also negatively correlated with the average median viewing distance (*r*_median distance_ = −0.524, *p* = 0.045).

In summary, while our measures of viewing distance are arguably noisy, our findings suggest the strong mediating role of the painting style, with the pattern of results obtained for the non-Indigenous paintings similar to that reported by Carbon (2017). The significant negative correlation between the amplitude spectrum slope and mean viewing distance across all paintings seem to suggest that the participants tend to move away from the patterns that have greater presence of high spatial frequency information and/or greater degree of spatial variegation. The negative correlation between Shannon entropy and viewing distance for the Indigenous paintings may be related to the relationship between “amount of information” and visual interest, however, in the absence of any direct psycho-physical measures of the components of aesthetic experience (such as visual interest), this assertion remains speculative.

#### Analysis of Gaze Metrics in the Museum

**FIGURE 7:**
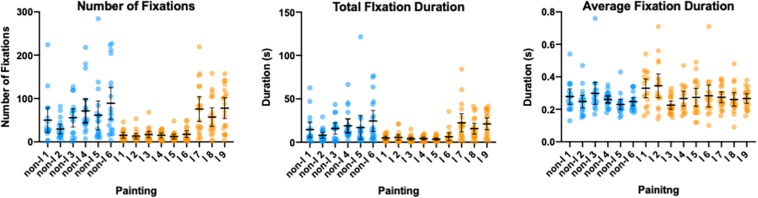
Number of fixations **(left panel)**, total fixation duration **(middle panel)** and average fixation duration **(left panel)** shown per each painting. Each dot represents data from one participant with the mean with 95%CI for each painting superimposed in black color. The data corresponding to the non-Indigenous and Indigenous paintings shown in blue and orange color, respectively. The paintings in each group are ordered from the smallest to the largest in area for that group. The error bars represent 95% CI.

For each participant we determined the total number of fixations (NF), total duration of fixations (TFD) and average fixation duration for each painting viewed. These data are shown per painting for the three gaze pattern metrics in Figure 7. Each symbol represents one participant’s data for a given painting. The data corresponding to the non-Indigenous and Indigenous paintings are shown in blue and orange colors, respectively (successive paintings in each category are ordered by the area from the smallest to the largest).

On average, participants made 44.24 fixations per painting (median = 42.9; *SD* = 21.07), with an average total fixation duration of 12.44 s (median = 11.24; *SD* = 7.42), and an average length of 0.270 ms (median = 0.254; *SD* = 0.005). To test whether there were statistically significant differences in the gaze measures between different paintings we performed separate mixed-effect models (REML) with Paintings (15 levels) as a repeated measures factor. These analyses revealed significant main effect of Paintings for the number of fixations [*F*_(4.87, 82.13)_ = 9.371, *p* < 0.001], the total fixation duration [*F*_(4.37, 73.68_ = 6.200, *p* < 0.001], as well as the average fixation duration [*F*_(4.97, 83.77_ = 3.207, *p* = 0.011]. To explore whether there was a systematic difference in the three eye gaze measures across the two painting style categories, we averaged these metrics across all non-Indigenous and Indigenous paintings, respectively. Paired t-tests revealed significant difference in the number of fixations (*t*_18_ = 3.074, *p* = 0.006) and the total fixation duration (TFD) (*t*_18_ = 2.263, *p* = 0.036) between the two groups of paintings: the non-Indigenous artworks received higher number of fixations (59.18) with longer total fixation durations (16.22 s) compared with Indigenous artworks (33.14 fixations and 9.557 fixation duration). The difference in the average fixation duration (AFD) between the two groups was not significant (*t*_18_ = 1.065, *p* = 0.309).

It is likely that these differences are related to the aforementioned general relationship between the painting size and the proportion of visitors. It seems that not only are the smaller paintings less likely to be visited at all, but, even when they are, they receive fewer fixations and are not looked at for as long as the larger paintings.

#### Image Statistics as Predictors of Eye Gaze Behavior

To examine the extent to which image statistics measures can be used to predict variance in eye gaze measures we performed three separate multiple regression analyses (enter method) with the number of fixations, total fixation duration and average fixation duration as dependant variables with the area, amplitude spectrum slope, fractal dimension and Shannon entropy as predictors in each case. These analyses show that the area (β = 0.799, *t* = 7.025, *p* < 0.001), fractal dimension (β = 0.643, *t* = 3.11, *p* < 0.011) and amplitude spectrum slope (β = 0.556, *t* = 2.752, *p* < 0.020) were significant predictors of number of fixations [*F*_(4, 10)_ = 18.556, *p* < 0.001]. The same was the case for the total fixation duration [*F*_(4, 10)_ = 13.638, *p* < 0.001] with the area (β = 0.762, *t* = 5.860, *p* < 0.001), fractal dimension (β = 0.667, *t* = 2.83, *p* < 0.018) and amplitude spectrum slope (β = 0.548, *t* = 2.376, *p* < 0.039) as significant predictors. None of the image statistics measures were significant predictors of the average fixation duration [*F*_(4, 10)_ = 0.858, *p* = 0.521].

### Laboratory Conditions

#### Analysis of Gaze Metrics in the Museum

**FIGURE 8:**
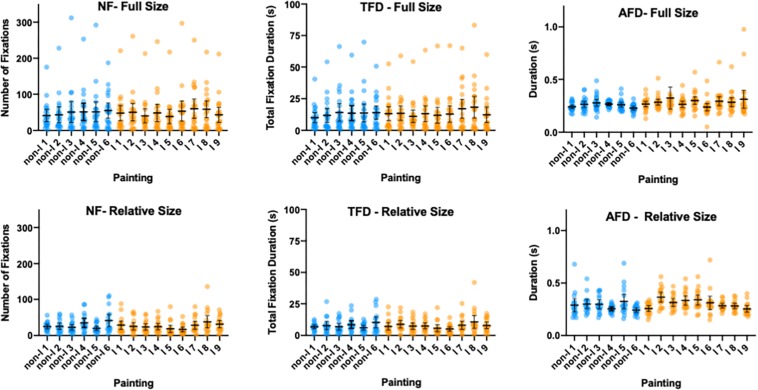
Number of fixations **(left panels)**; total fixation duration **(middle panels)**; and average fixation duration **(left panels)** shown for each painting in the full-size screen condition **(top row)** and the relative size screen condition **(bottom row)**. Each dot represents data from one participant with the mean with 95% CI for each painting superimposed in black color. The data corresponding to the non-Indigenous and Indigenous paintings shown in blue and orange color, respectively. The paintings in each group are ordered from the smallest to the largest in area for that group. The error bars represent 95% CI.

As with the gaze data in the museum condition, the total number of fixation (NF; left panels), total duration of fixations (TFD; middle panels) and average fixation duration (AFD, right panels) for each painting are shown in Figure 8. The top row shows the data from the full-screen condition in which the longest dimension for each painting (either horizontal or vertical) was made to till the screen while the bottom row shows the data from the relative-screen condition in which the relative size differences were preserved between the paintings. Each symbol represents data from one participant and the data corresponding to the non-Indigenous and Indigenous paintings are shown in blue and orange colors, respectively (successive paintings in each category are ordered by area from the smallest to the largest). In order to test whether there were significant differences in three gaze metrics between the two on-screen presentation conditions we performed two way mixed-effect models (REML) with paintings (15 levels) as within and on-screen presentation condition (full-size vs. relative-size) as between subject factors, respectively.

##### Number of fixations.

The mean number of fixations per painting was 49.9 in the full-screen condition, compared to the mean of 27.03 for the relative size condition. The two-way mixed-effect model (REML) revealed the significant main effect of paintings [*F*_(6.235, 230.7)_ = 5.856, *p* < 0.001] and the significant paintings x on-screen presentation condition interaction [*F*_(14, 518)_ = 2.375, *p* = 0.003]. However, the main effect of on-screen presentation condition was not significant [*F*_(1, 37)_ = 2.985, *p* = 0.0924].

##### Total fixation duration.

The mean fixation duration per painting was 13.39 s in the Full-screen condition, compared to the mean of 7.44 s for the relative size condition. The two-way mixed-effect model (REML) revealed the significant main effect of paintings [*F*_(7.043, 260.6)_ = 5.018, *p* < 0.001]. The main effect of on-screen presentation condition was not significant [*F*_(1, 37)_ = 2.947, *p* = 0.0944], nor was the paintings’ x on-screen presentation condition interaction [*F*_(14, 518)_ = 1.599, *p* = 0.0751].

##### Average fixation duration.

The mean fixation duration per painting was 0.273 s in the full-screen condition, compared to the mean of 0.296 s for the relative size condition. The two-way mixed-effect model (REML) revealed the significant main effect of paintings [*F*_(3.585, 132.4)_ = 3.712, *p* < 0.009], as well as the significant paintings x on-screen presentation condition interaction [*F*_(14, 517)_ = 2.567, *p* = 0.0014]. The main effect of on-screen presentation condition was not significant [*F*_(1, 37)_ = 1.533, *p* = 0.223].

#### Image Statistics as Predictors of eye Gaze Metrics

As for the in-museum condition, in order to examine whether the image statistics measures can be used to predict variance in eye gaze metrics we performed three separate multiple regressions (enter method) with the area, amplitude spectrum slope, fractal dimension and Shannon entropy as predictors.

##### Full-size on-screen presentation condition.

Even though there were no major changes in the area, or the visual angle subtended by different paintings in this condition, the area was kept as one of the predictors to keep the parameters of regression analyses comparable across different presentation conditions. The results show that none of the image statistics measures were significant predictors of the average number of fixations [*F*_(4, 10)_ = 1.212, p = 0.365) and the same was observed for the total fixation duration [*F*_(4, 10)_ = 3.309, *p* = 0.057]. However, the Shannon Entropy (b = −0.861, *t* = −5.589, *p* < 0.001) was the significant predictor of the average fixation duration [*F*_4, 10)_ = 18.556, *p* < 0.001]. The same was the case for the total fixation duration [*F*_(4, 10)_ = 10.413, *p* < 0.001]. Negative standardized b coefficient suggests that the average fixation length was shorter for the paintings with higher entropy values.

##### Relative-size on-screen presentation condition.

The results show that none of the image statistics measures were significant predictors for the number of fixations [*F*_(4, 10)_ = 2.430, *p* < 0.116], fixation duration [*F*_(4, 10)_ = 1.171, *p* = 0.380], or average fixation length [*F*_(4, 10)_ = 2.193, *p* = 0.143].

### Comparison Between Museum and On-Screen Conditions

**FIGURE 9:**
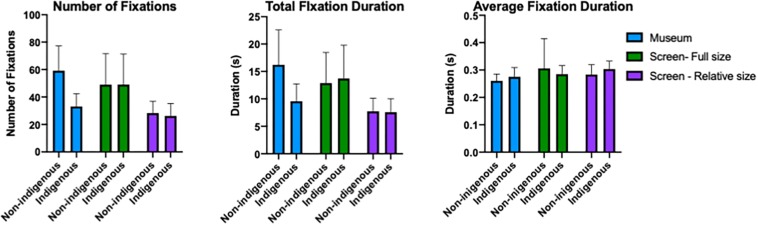
Mean number of fixations, total fixation duration and average fixation length for Indigenous and non-Indigenous paintings in the three presentation conditions (museum; screen, full size; screen, relative size). The error bars represent 95% CI.

To compare the gaze metrics between all three presentation conditions we performed two-way mixed-effect ANOVA with painting style (non-Indigenous, Indigenous) as a repeated measures and presentation condition (museum; screen, full size; screen, relative size) as a between-subject factor. The mean number of fixations, total fixation duration and average fixation length for each presentation condition and the painting style groups are depicted in Figure 9.

#### Number of Fixations

The main effect of presentation condition did not reach significance [*F*_(2, 55)_ = 2.080, *p* = 0.1347], but the interaction between the presentation condition and painting style was significant [*F*_2, 55)_ = 8.358, *p* < 0.001]. The main effect of painting style was also significant [*F*_1, 55)_ = 10.35, *p* = 0.002]. The significant interaction indicates that the difference in number of fixations for the two painting styles was pronounced only in the museum condition.

#### Total Fixation Duration

The main effects of presentation condition [*F*_(2, 55)_ = 1.927, *p* = 0.1553] and painting style [*F*_(1, 55)_ = 3.924, *p* < 0.053] did not reach significance, but the interaction between the presentation condition and painting style was significant [*F*_(2, 55)_ = 5.532, *p* = 0.006]. Analogous to the pattern with number of fixations, the difference in the total fixation duration for the two painting styles was visible only in the museum condition.

#### Average Fixation Duration

The main effect of painting style was significant [*F*_(1, 55)_ = 9.125, *p* < 0.004] such that overall the average fixation duration for the Indigenous paintings was longer than for the non-Indigenous paintings. This trend seems to be more pronounced in the museum and in the on-screen relative size conditions, but the interaction between painting style and presentation condition was not significant [*F*_(2, 55)_ = 0.349, *p* = 0.707]. The main effect of presentation condition was not significant [*F*_(2, 55)_ = 1.237, *p* < 0.298].

### Fixations Location Heatmaps for Artworks in Different Viewing Contexts

**FIGURE 10:**
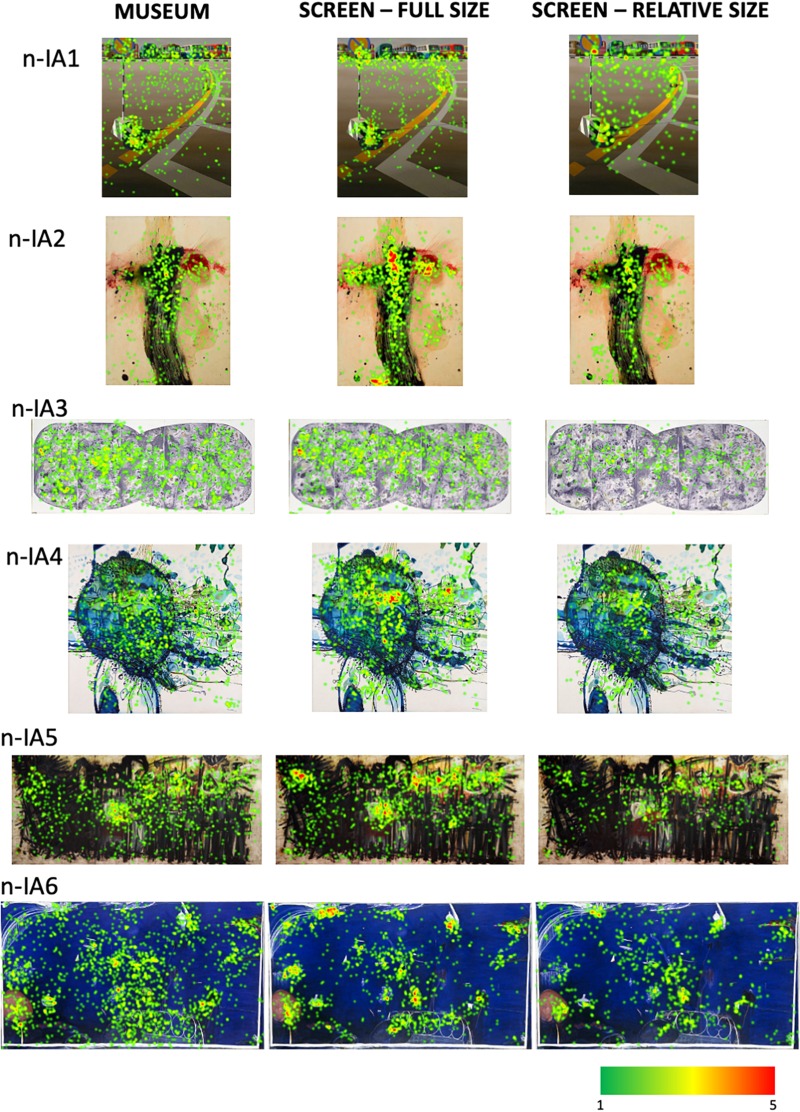
The heatmaps of total fixation counts for the non-Indigenous Australian paintings in the three viewing conditions. See Supplementary Material for bigger, higher-quality versions of these heatmaps.

**FIGURE 11:**
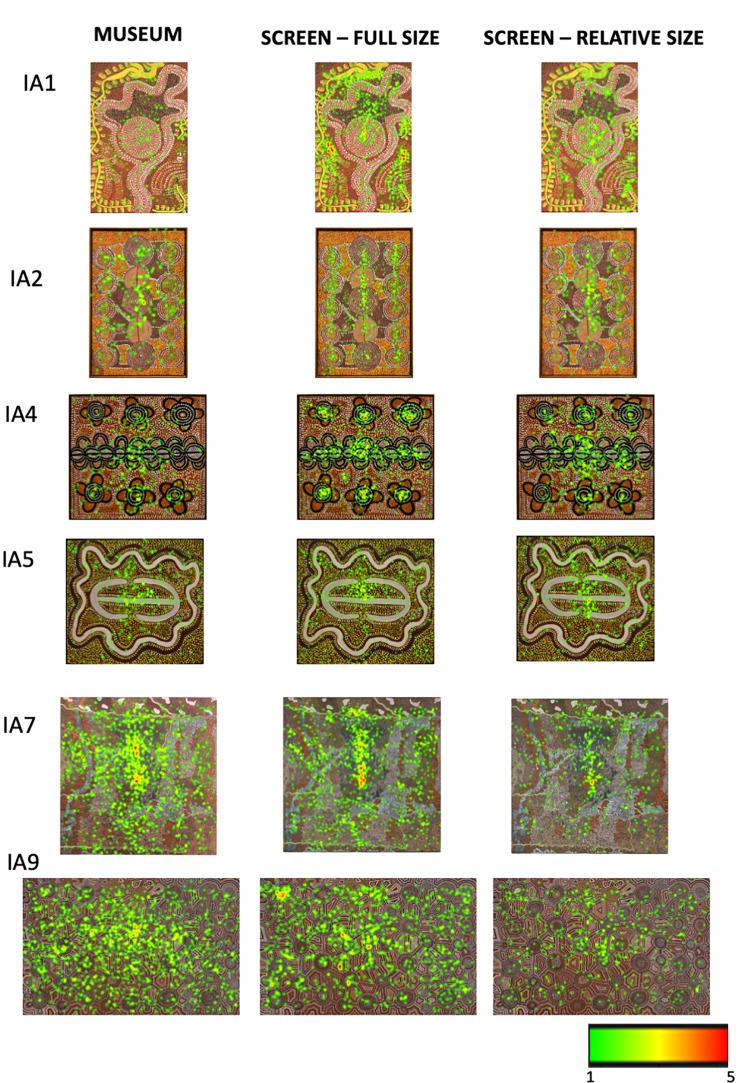
The heatmaps of total fixation counts for some of the Indigenous Australian paintings in the three viewing conditions. See Supplementary Material for the heatmaps for all Indigenous Australian paintings.

In order to provide further qualitative and quantitative insights in viewing behavior across the three different contexts used in our study, we have generated heatmaps of total fixations for each artwork in each condition. They are summarized for the non-Indigenous and some of the Indigenous paintings in Figures 10, 11, respectively. The bigger and higher quality heatmaps for each painting can be found in Supplementary Materials.

**FIGURE 12:**
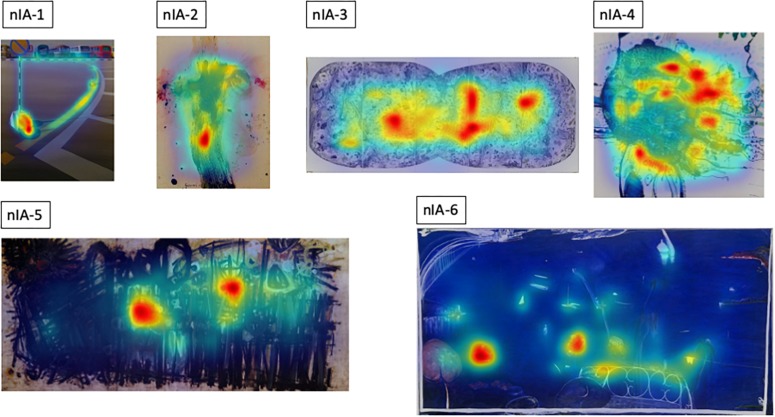
Graph-based Visual Saliency maps (Harel et al., 2007) for the non-Indigenous Australian paintings.

**FIGURE 13:**
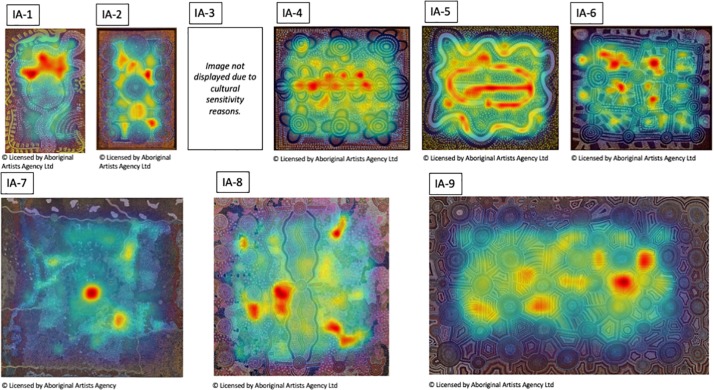
Graph-based Visual Saliency maps (Harel et al., 2007) for the Indigenous Australian paintings.

To provide a low-level visual baseline we computed an objective, visual saliency map of each image using the Graph-Based Visual Saliency method (Harel et al., 2007) which are shown in Figures 12, 13. They are computer-generated saliency analyses of the original images’ low-level visual features (e.g., luminance, color, orientation, contrast, edge, etc.) represented as a heat map, the warmest color indicating the areas of highest image-based saliency. For most, if not all of the images, they seem in a good agreement with the fixation heatmaps though we do not have any quantitative measures of comparison.

Though differing in the overall number of fixations, the spatial layout of fixated locations is remarkably similar across the three different contexts in which the artworks were viewed from different distances, for different durations, at different visual angles and in different mediums. This similarity is consistent with the two-stage model proposed by Locher (1996) according to which, exploration of a painting starts with a rapid global survey of the pictorial field to acquire an initial structural gist of the composition, followed by the second phase of visual scrutiny of interesting pictorial features.

### Interindividual Similarity in Viewing of Artworks in Three Conditions

used a ScanMatch MatLab toolbox (Cristino et al., 2010) to quantify the similarity in fixation sequences between different participants in each viewing condition. In ScanMatch the individual fixation sequences are spatially and temporally binned to create a sequence of letters that retains fixation location, time and order information. The algorithm then uses the Needleman-Wunsch sequence alignment procedure to compare the coded sequences and, based on the inverse Euclidian distance of each fixations pair, calculates a similarity score (0–1). For each painting, we calculated similarity scores for all pairwise combinations between participants who viewed the painting in each of the three conditions. Based on the pairwise similarity scores for all possible pairings between participants, we calculated an average similarity score for each painting in three conditions.

## Discussion

In this study we investigated the viewing behavior of visitors (*N* = 19) freely viewing 15 paintings in a room containing 20th-century Australian artworks at the Art Gallery of New South Wales. We examined how aspects of viewing behavior including viewing distance in the gallery condition and eye gaze measures such as fixation count, total fixation duration and average fixation duration are affected by the artworks’ physical characteristics of size and image statistics properties such as Fourier amplitude spectrum, fractal dimension and entropy. Given the diversity of artworks in the collection, we have also considered the painting style, Indigenous Australian vs. non-Indigenous Australian as an additional characteristic of interest in our study.

On average, the participants spent 8.68 min in the gallery room and looked at 94% of the paintings. We observed a significant correlation between the average proportion of participants who viewed a painting and the painting’s physical size. This relationship was more pronounced for the contemporary non-Indigenous compared to Indigenous paintings. The average mean viewing distance across all paintings was 1.37 m, with the shorter average viewing distance for non-Indigenous (1.24 m) compared to Indigenous paintings (1.4 m). There was also a positive relationship, although only for the non-Indigenous paintings, between the painting area and viewing distance, as observed by Carbon (2017). With Indigenous paintings, there was a negative relationship between viewing distance and Shannon entropy coefficient. In addition, across all paintings, the slope of the Fourier amplitude spectrum was negatively correlated with the median viewing distance. In summary, while our measures of viewing distance are arguably noisy, our findings suggest the strong mediating role of the painting style on the viewing distance. The significant negative correlation between the amplitude spectrum slope and mean viewing distance across all paintings seem to suggest that the participants tend to move away from the patterns that have greater presence of high spatial frequency information and/or greater degree of spatial variegation.

Our eye gaze measures in the gallery condition revealed that on average participants made 44.24 fixations per painting with an average total fixation duration of 12.44 s and fixation length of 0.270 ms. Although the total fixation duration observed in our study is shorter than the average viewing times reported in earlier studies (Locher et al., 1999, 2001; Smith and Smith, 2001; Brieber et al., 2014, 2015; Carbon, 2017), our values include fixations only and do not reflect the total duration that the participants might have spent in front of paintings. There was also a significant effect of painting style with higher number of fixations and longer fixation durations for non-Indigenous compared to Indigenous paintings. We believe that these differences are related to the effects of painting size: not only are the smaller paintings less likely to be visited at all, but, seemingly, even when they are, they receive fewer fixations and are not looked at for as long as bigger paintings. Indeed, the multivariate regression analyses have revealed significant effect of area, fractal dimension and amplitude spectrum slope on both number of fixation and fixation duration.

However, when the same artworks were viewed in the laboratory, either scaled to fit most of the screen or to preserve their relative size as in the museum condition, none of the image statistics measures could be used to predict the average number of fixations and fixation duration. The only exception was Shannon entropy, which correlated negatively with the fixation duration and length, suggesting the shorter fixation duration and average length for paintings with higher entropy values.

Overall, when museum and on-screen presentation conditions were directly compared, our results reveal a strong interaction between presentation conditions and the effect of painting style and associated physical characteristics of artworks. We suggest that in order to be able to fully characterize the effect of presentation context in engaging with aesthetic objects, a finer grained analysis of their physical characteristics seem promising.

## Conclusion

In conclusion, our study indicate that individual paintings exert a strong influence of viewing behavior. Some of that influence can be attributed to the paintings’ physical and statistical image properties, especially when these properties coincide with differences in painting style. However, the experience of artworks in different contexts remains a complex question that requires a more robust and parametric manipulation of these factors. Concurrent measures of aesthetic experience should also be incorporated in future studies in this area.

## Data Availability Statement

The datasets generated for this study are available on request to the corresponding author.

## Ethics Statement

The studies involving human participants were reviewed and approved by the University of New South Wales Human Research Ethics Advisory Panel B (Arts, Humanities and Law) and Panel C (Psychology). The participants provided written informed consent to participate in this study.

## Author Contributions

VE-G, MG, SE, and BS contributed to the conception and design of the study. VG-E, MG, and SE contributed to the data collection at the AGNSW. VG-E and BS contributed to the data collection in laboratory and analyzed data. All authors contributed to the writing of the manuscript and approved its submission.

## Conflict of Interest

The authors declare that the research was conducted in the absence of any commercial or financial relationships that could be construed as a potential conflict of interest.
