# Supplementary material for: Viewing Art in Different Contexts
Source: Front Psychol. 2020 Apr 2;11:569. doi: 10.3389/fpsyg.2020.00569 (PMC7142233; doi:10.3389/fpsyg.2020.00569)

Supplementary Materials

Fixation Location Heatmaps for all artworks in the three different viewing conditions. Each panel shows the total number of fixations at each viewed location (green =1 fixation; red=5 fixations).

nIA-1 - Total Fixation Count

Museum

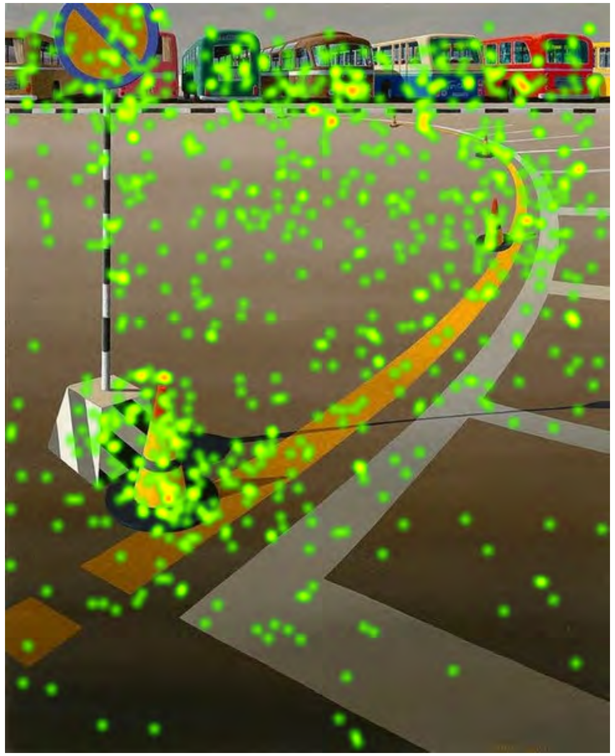

Screen – Full size

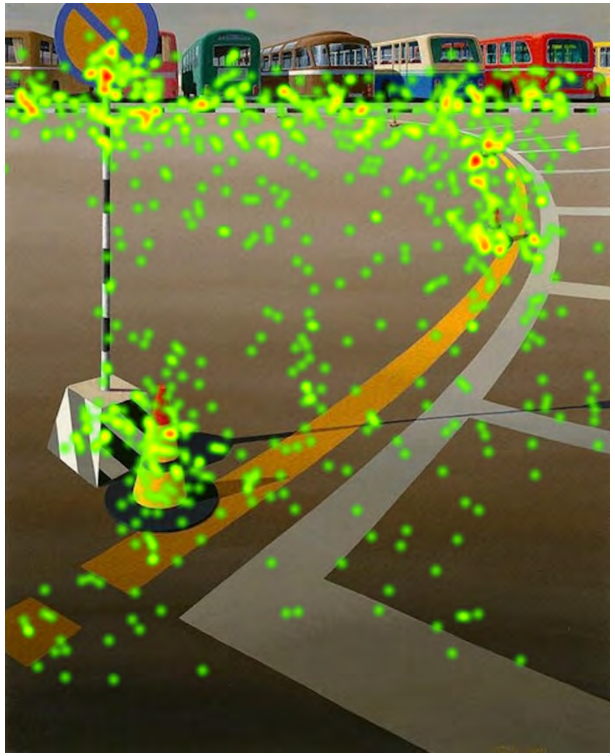

Screen – Relative Size

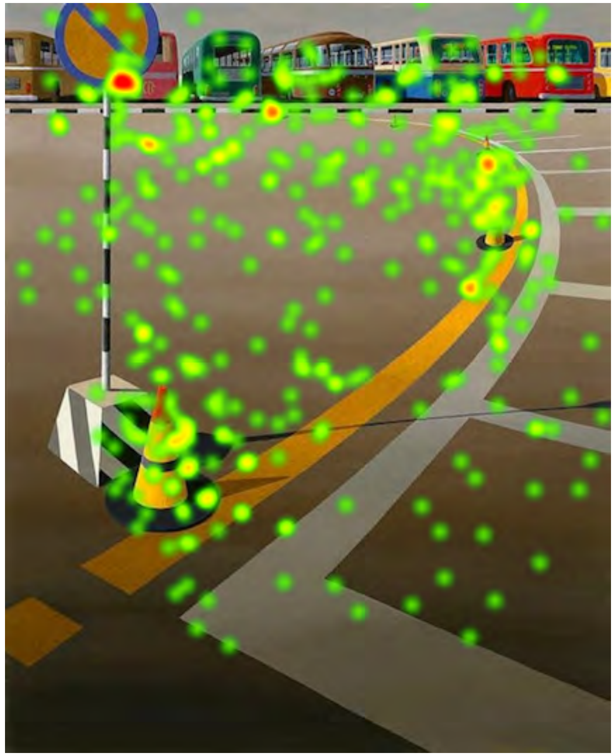

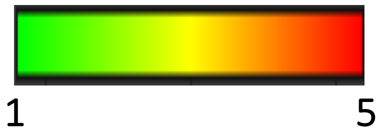

nIA-2 - Total Fixation Count

Museum

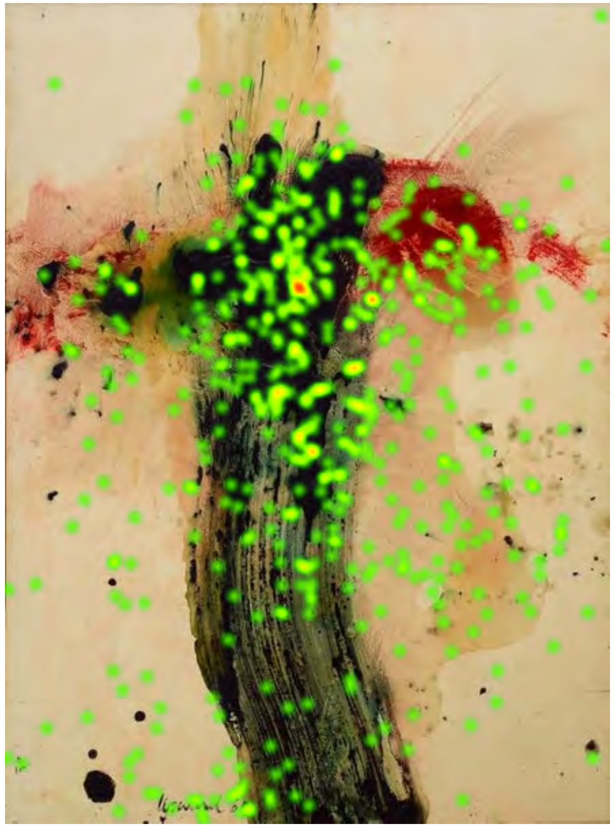

Screen – Full size

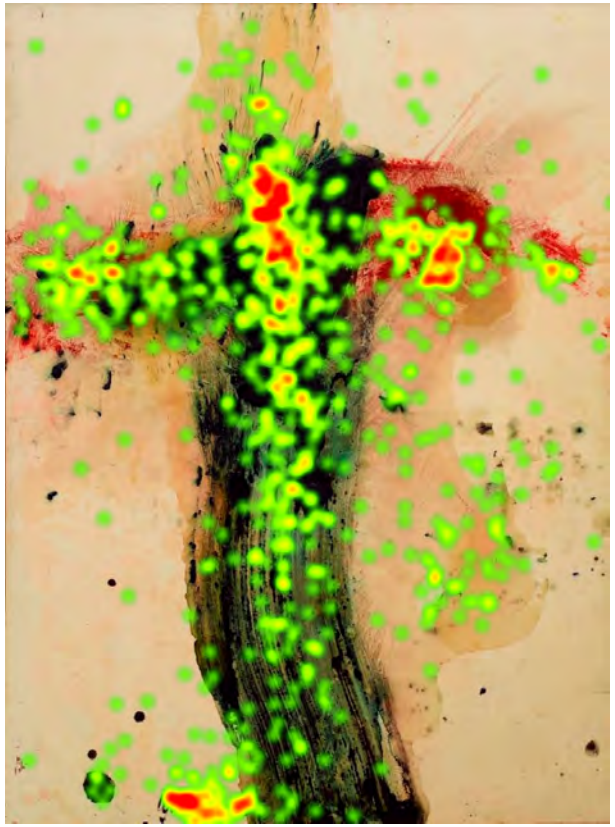

Screen – Relative Size

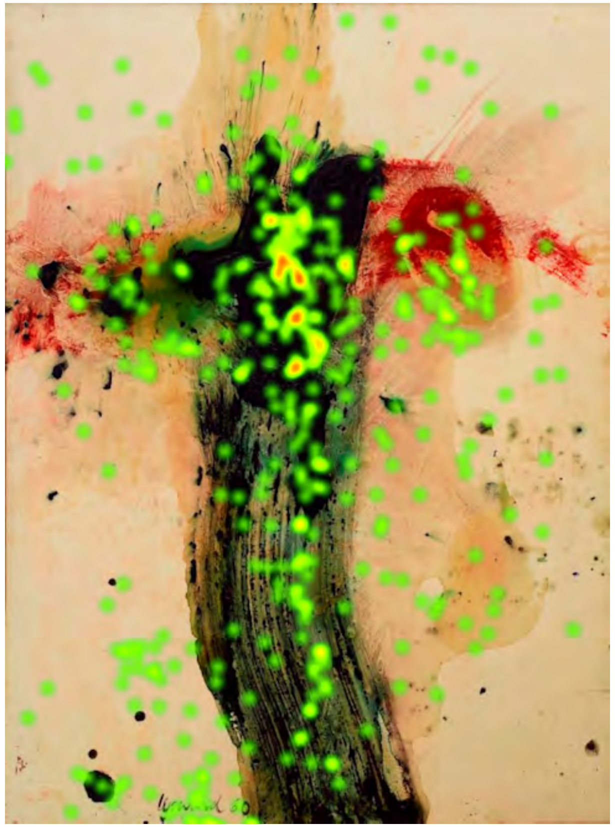

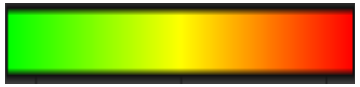

nIA-4 - Total Fixation Count

Museum

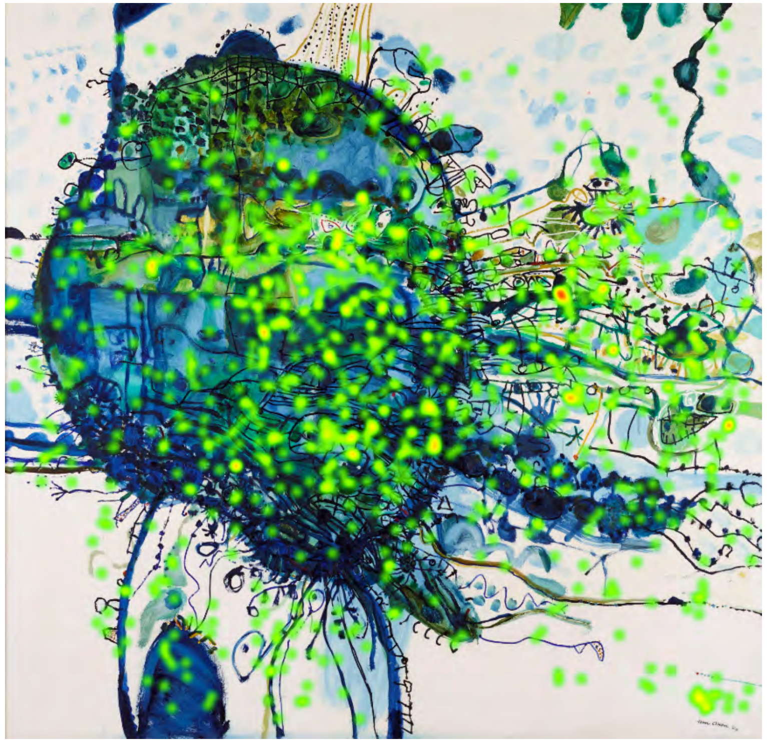

Screen – Full size

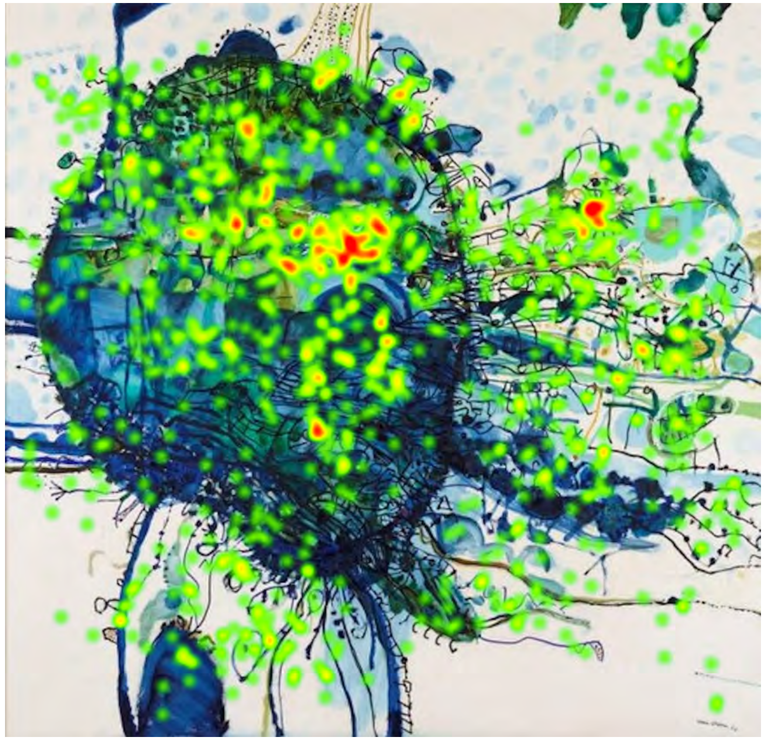

Screen – Relative Size

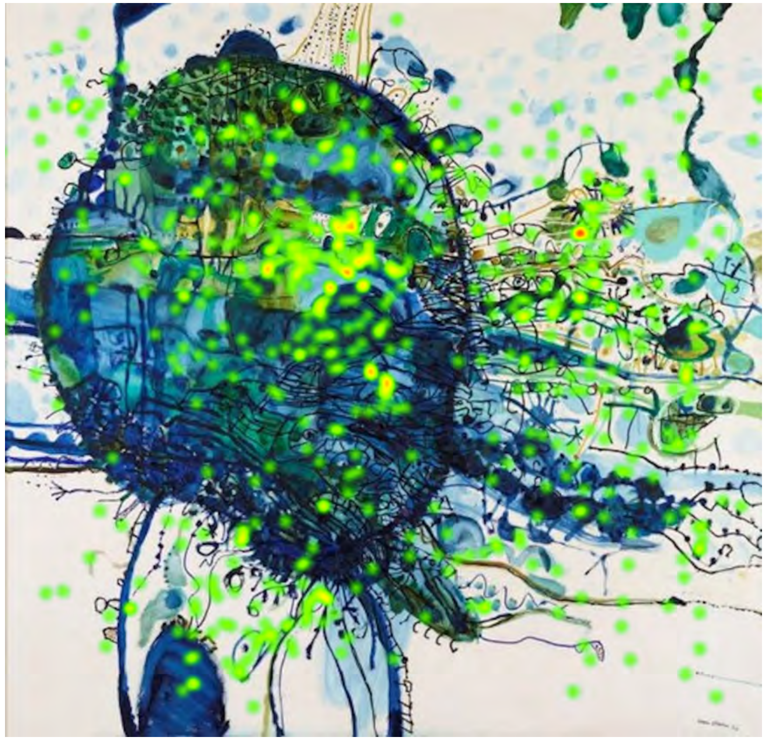

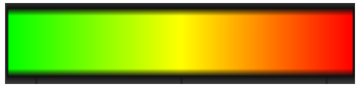

nIA-3 - Total Fixation Count

Museum

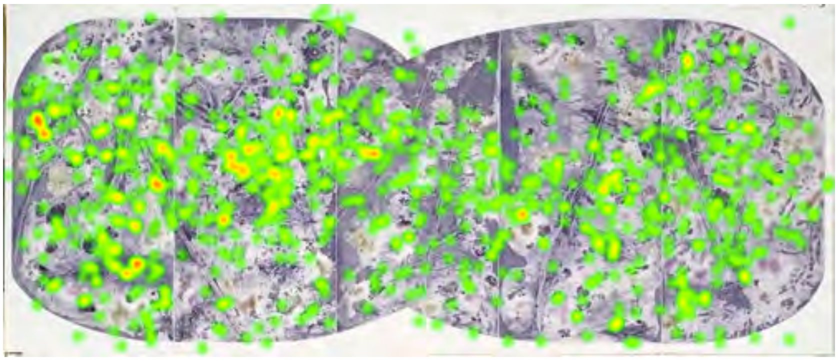

Screen – Full size

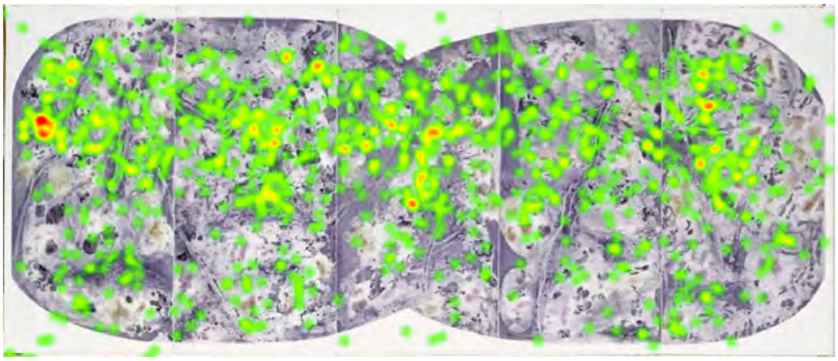

Screen – Relative Size

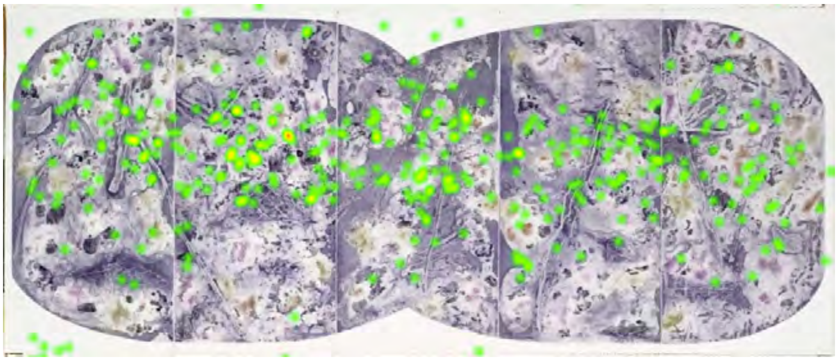

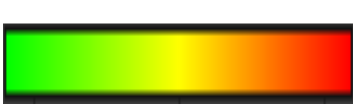

1 5

nIA-5 - Total Fixation Count

Museum

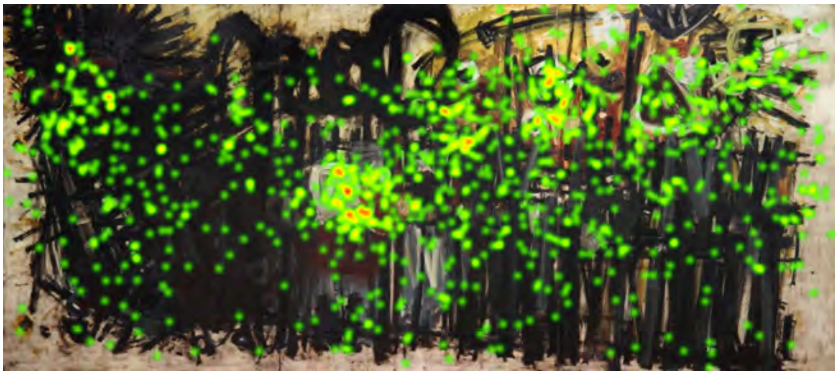

Screen – Full size

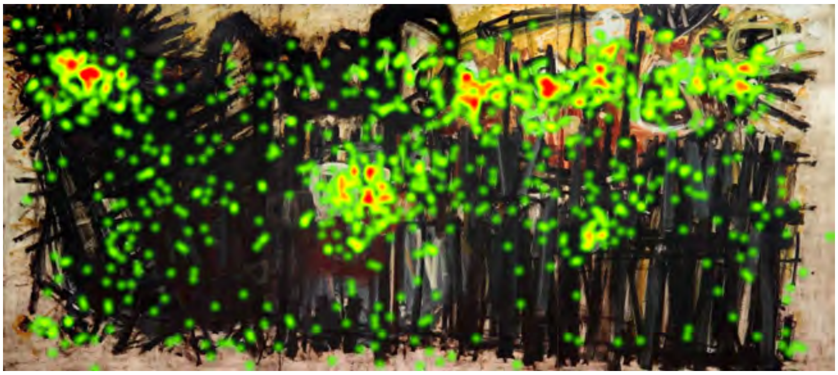

Screen – Relative Size

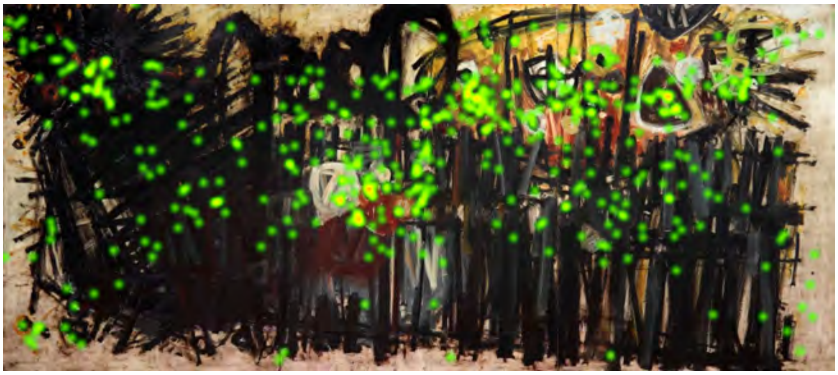

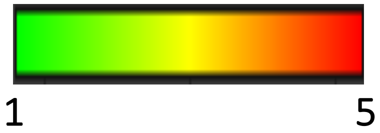

nIA 6 - Total Fixation Count

Museum

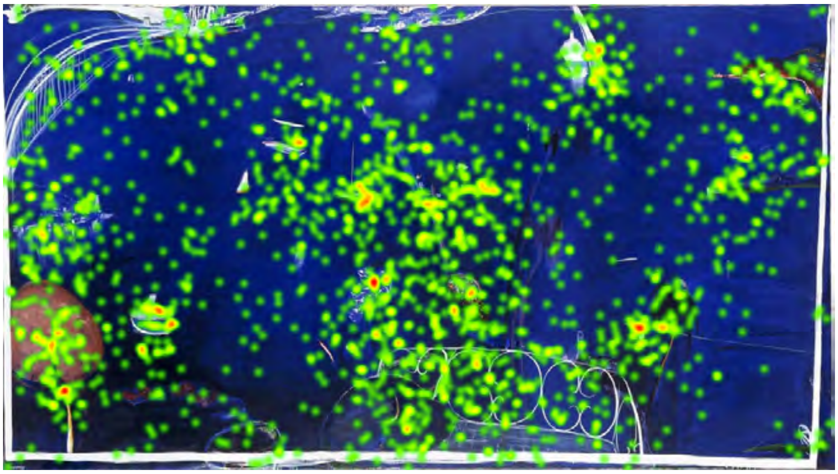

Screen – Full size

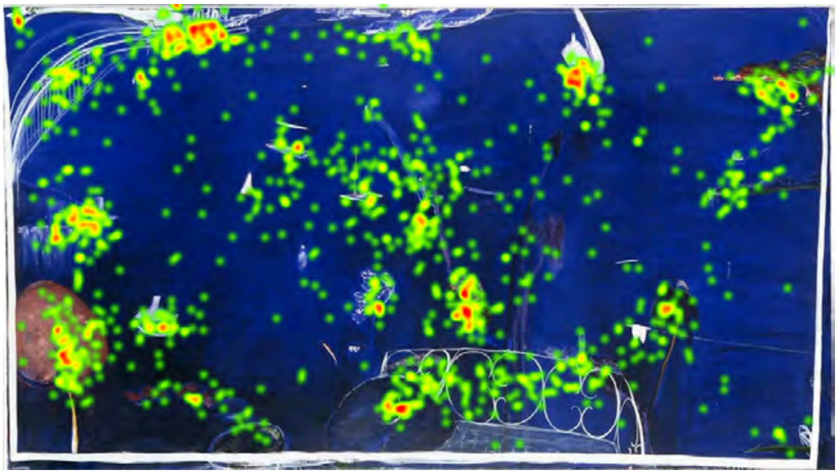

Screen – Relative Size

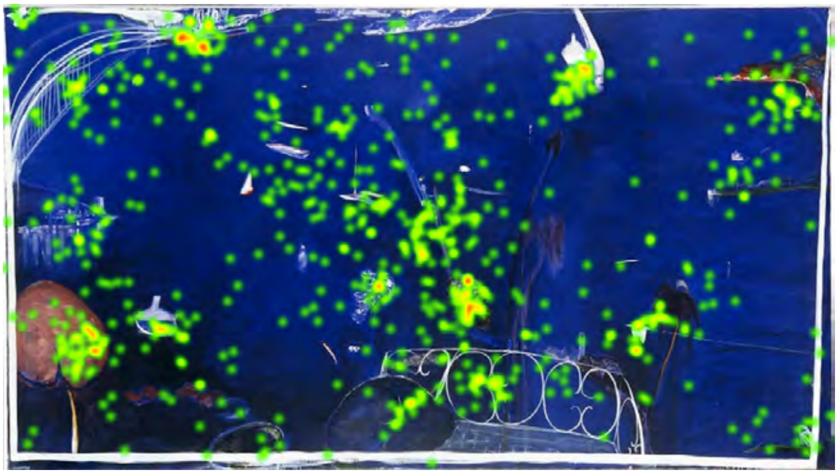

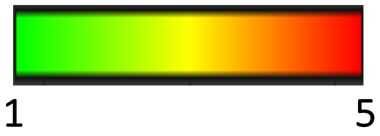

IA-1 - Total Fixation Count

Museum

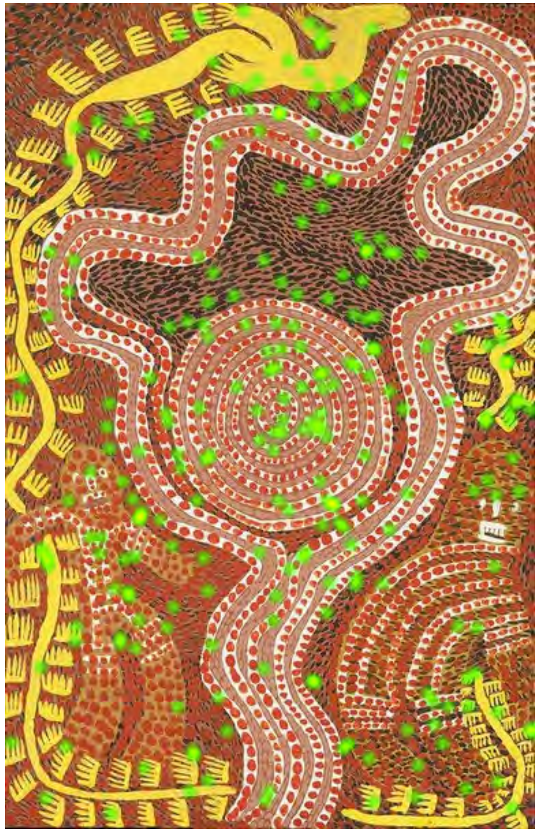

Screen – Full size

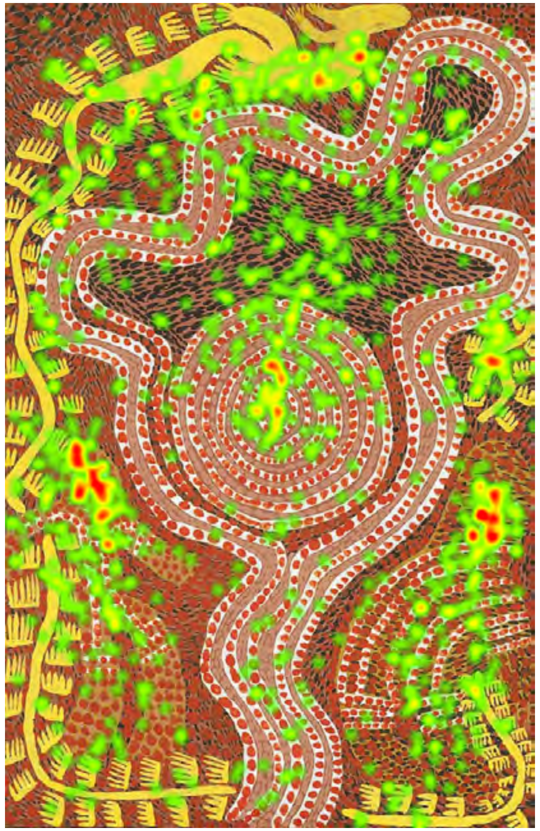

Screen – Relative Size

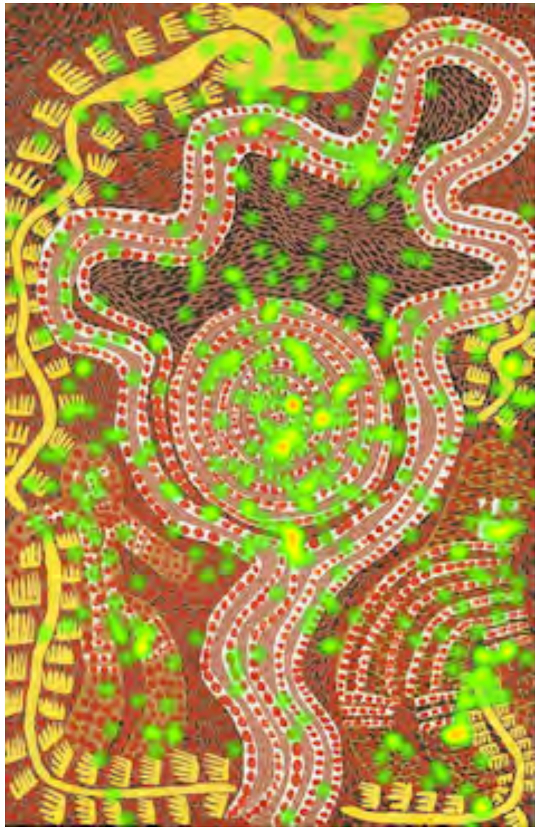

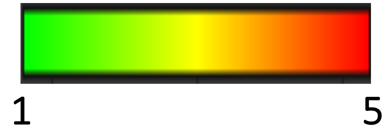

IA-2 - Total Fixation Count

Museum

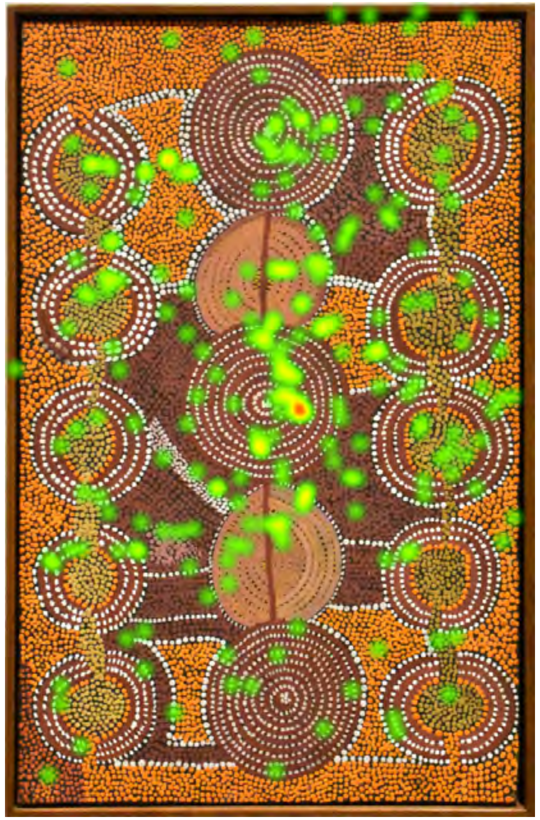

Screen – Full size

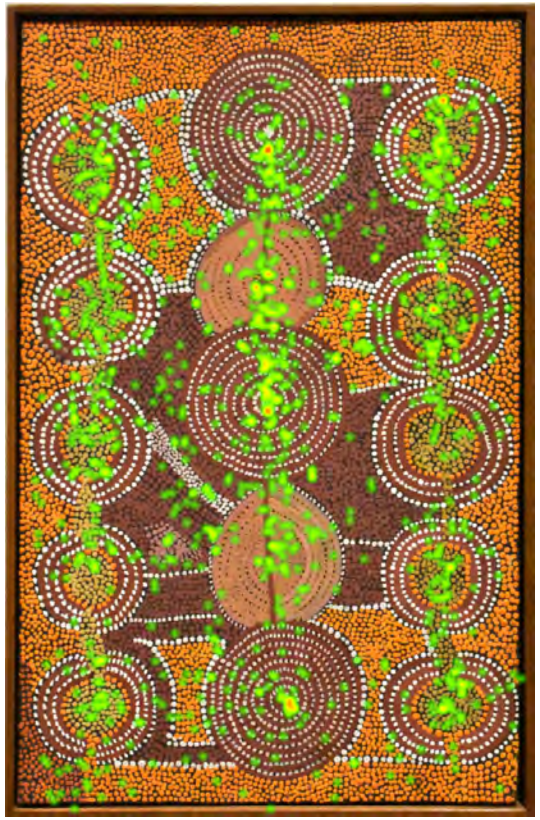

Screen – Relative Size

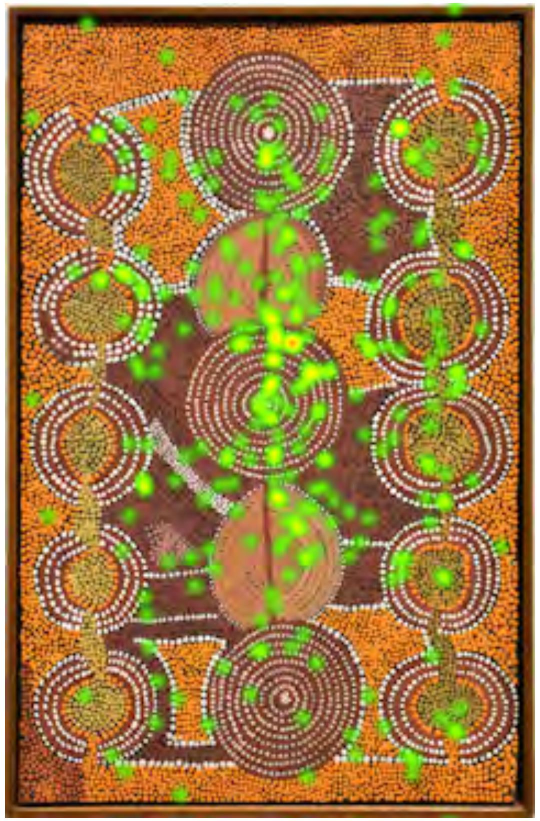

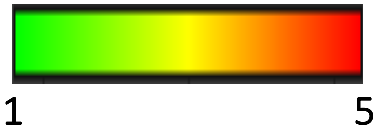

IA-3 - Total Fixation Count

Museum

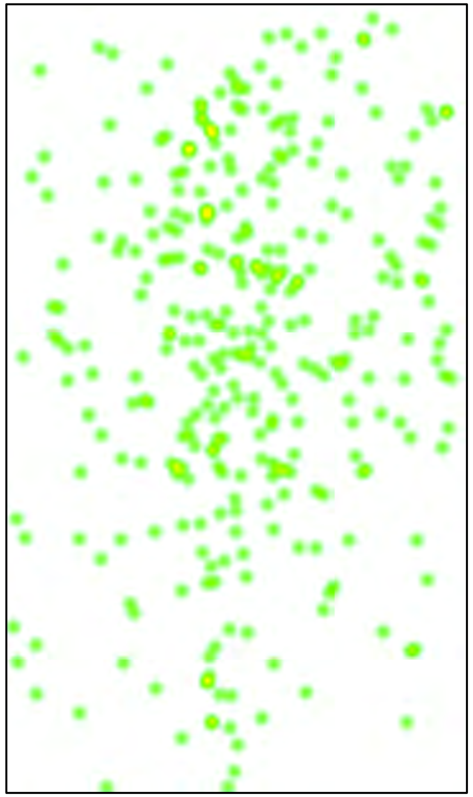

Screen – Full size

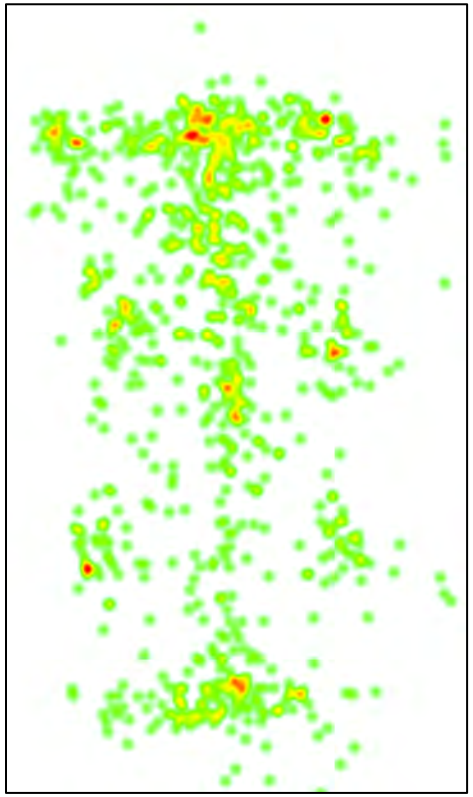

Screen – Relative Size

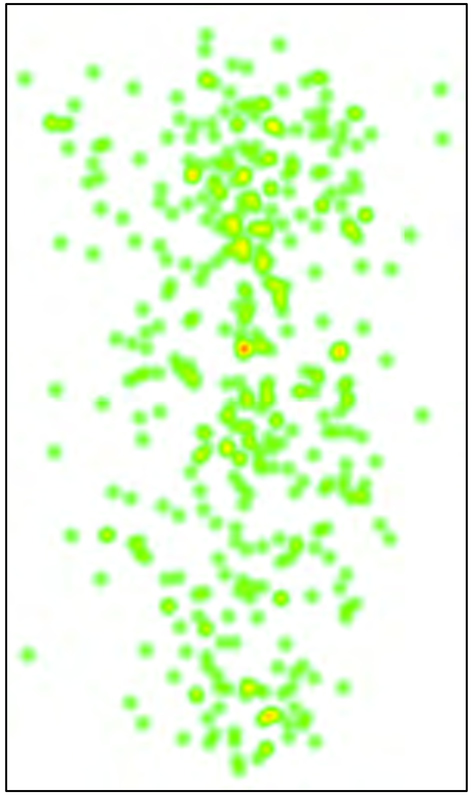

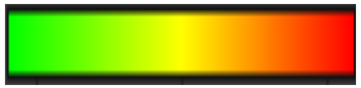

IA-4 - Total Fixation Count

Museum

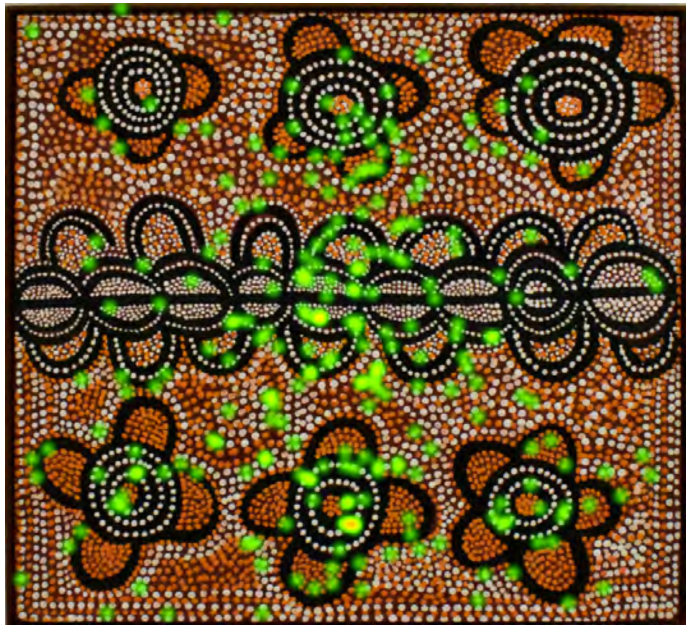

Screen – Full size

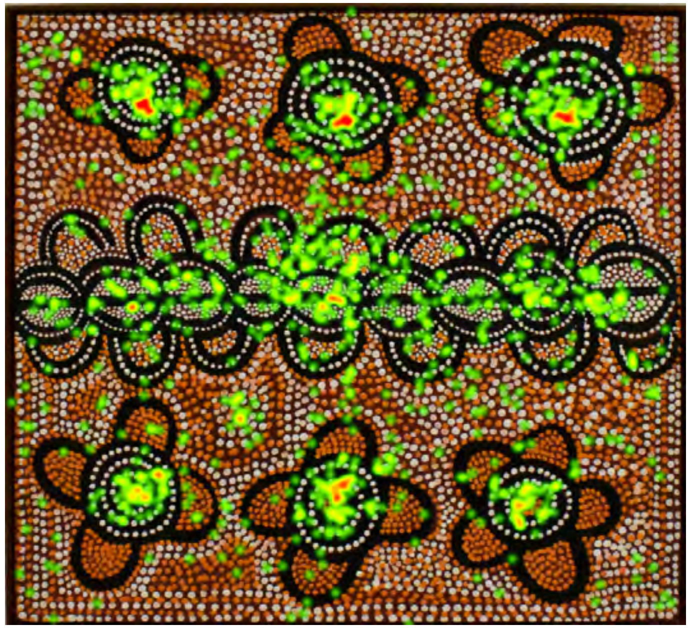

Screen – Relative Size

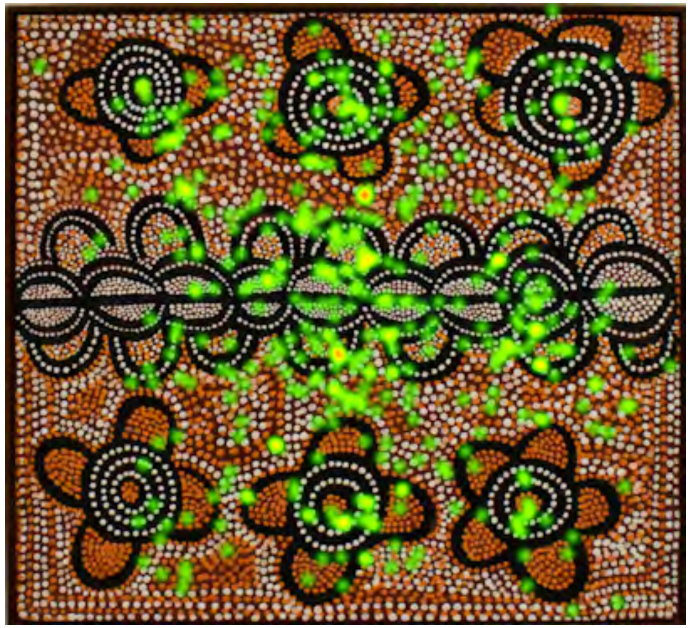

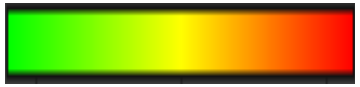

IA-5 - Total Fixation Count

Museum

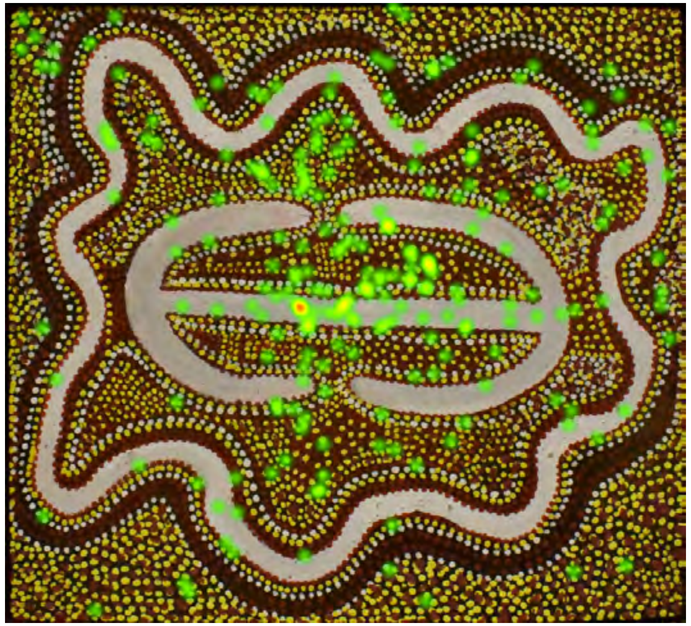

Screen – Full size

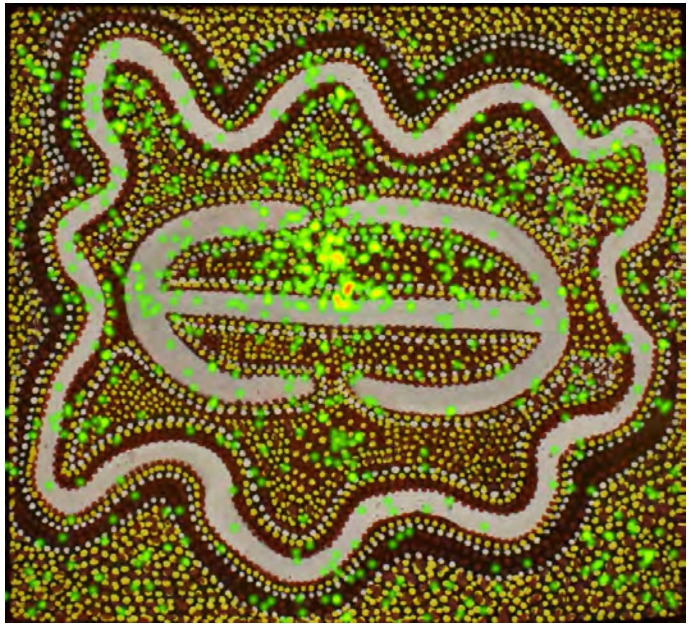

Screen – Relative Size

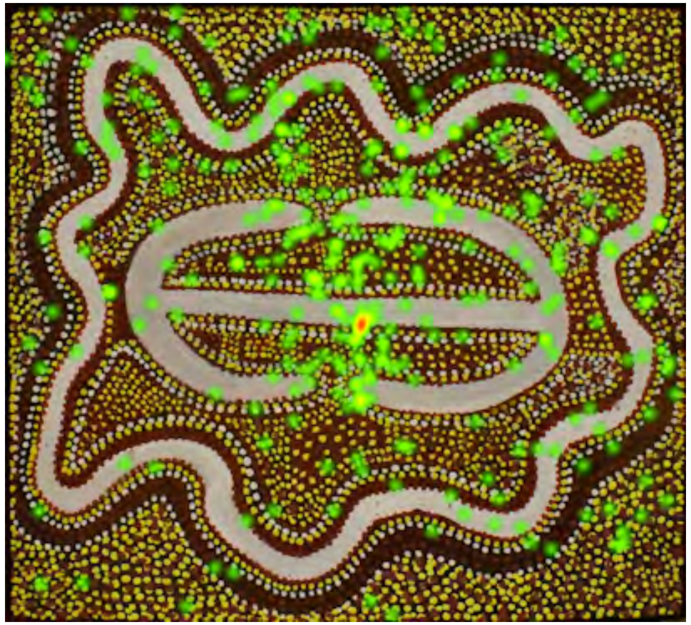

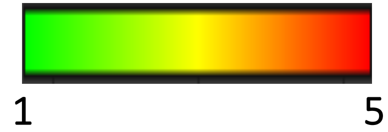

IA-6 - Total Fixation Count

Museum

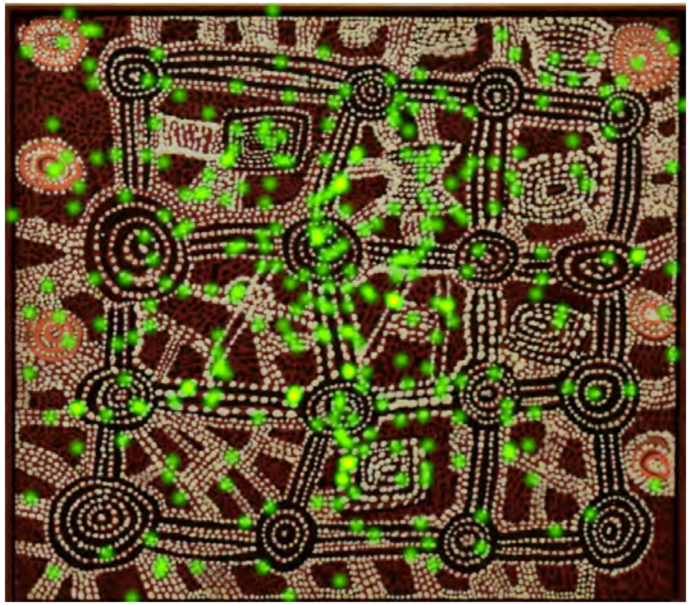

Screen – Full size

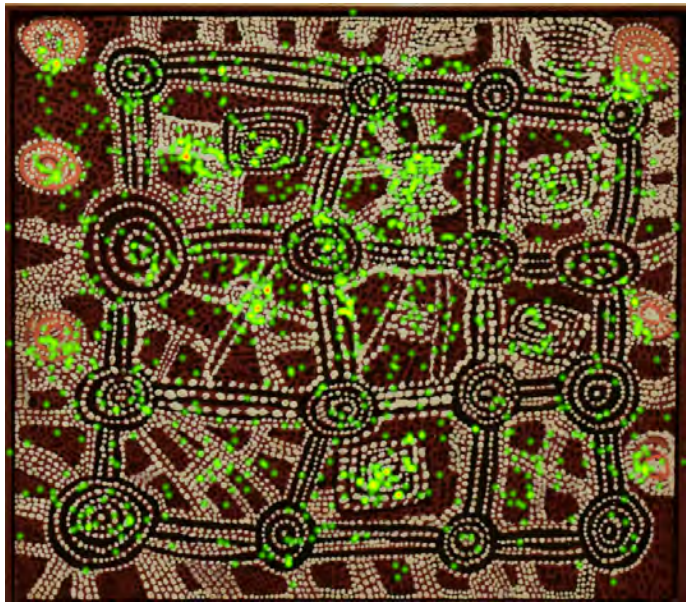

Screen – Relative Size

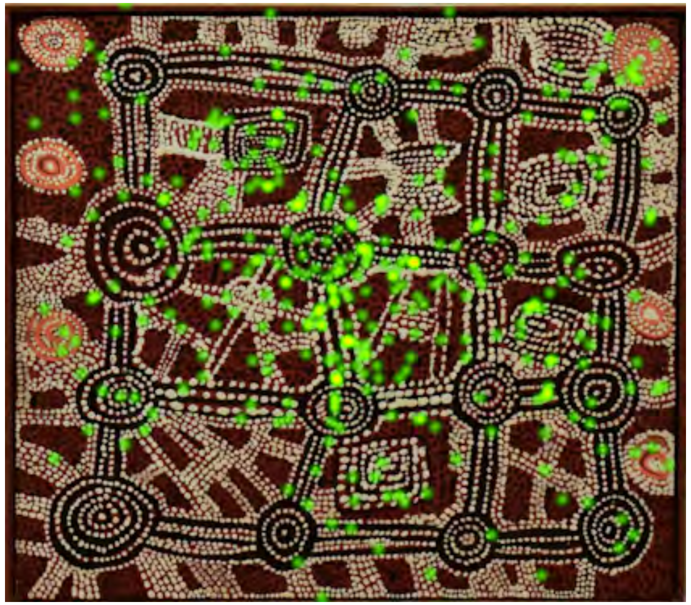

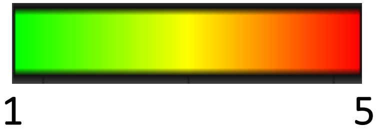

IA-7 - Total Fixation Count

Museum

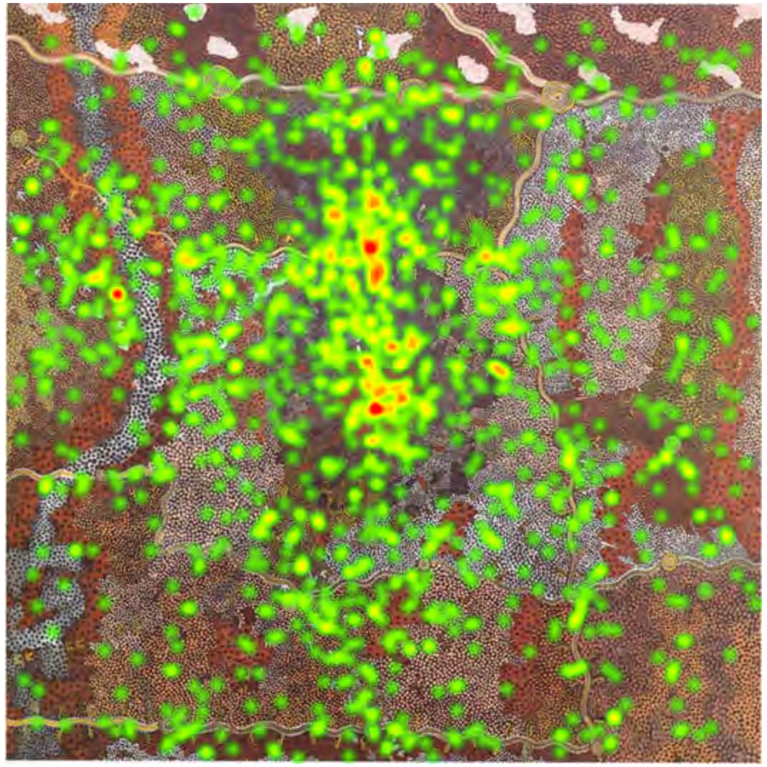

Screen – Full size

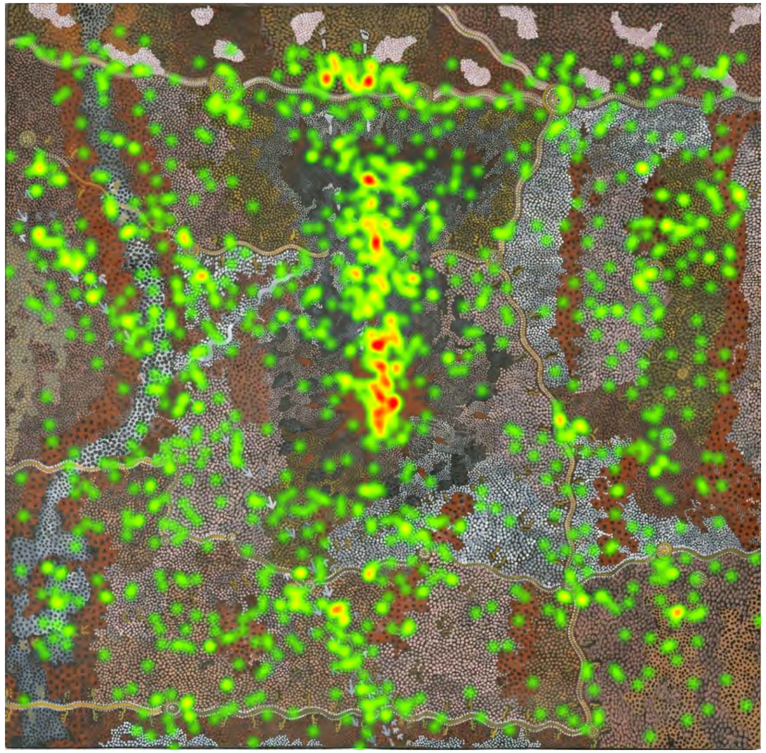

Screen – Relative Size

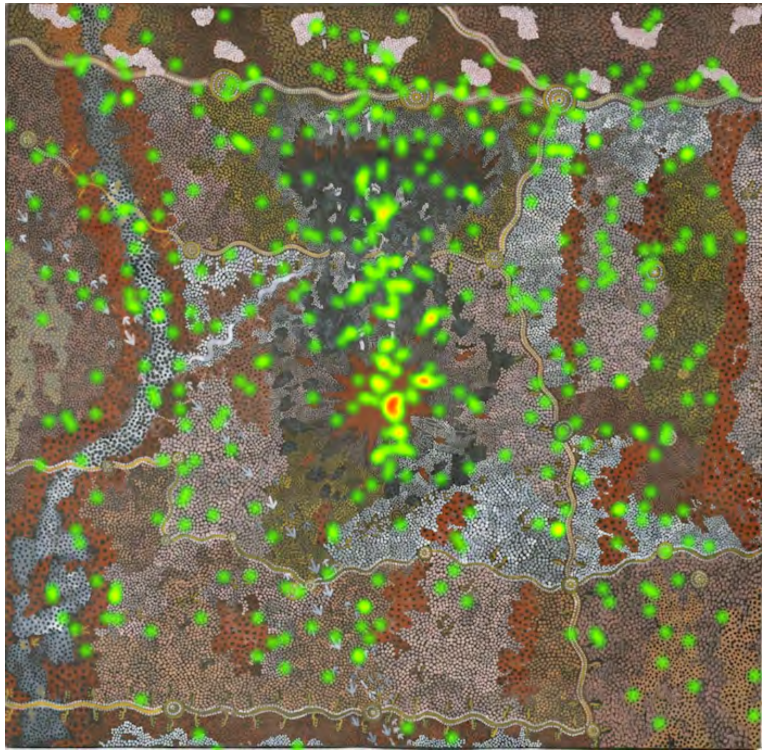

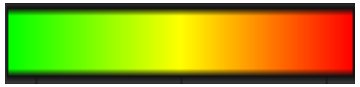

IA-7 - Total Fixation Count

Museum

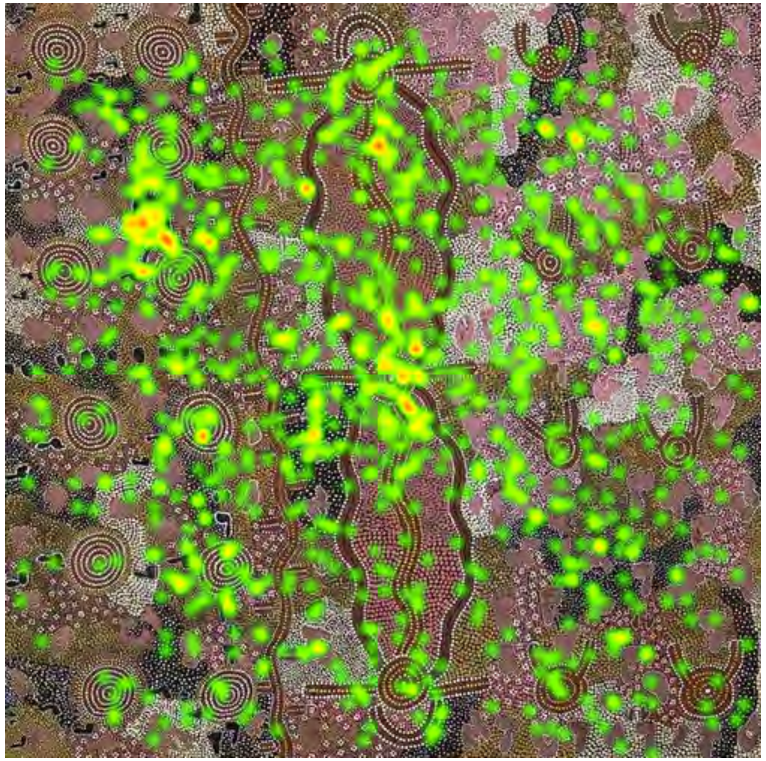

Screen – Full size

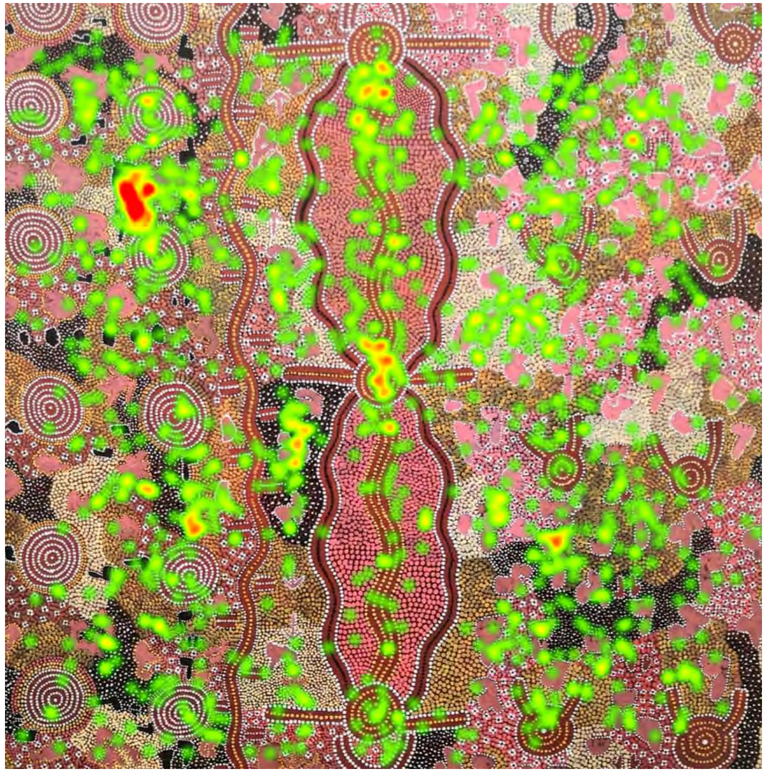

Screen – Relative Size

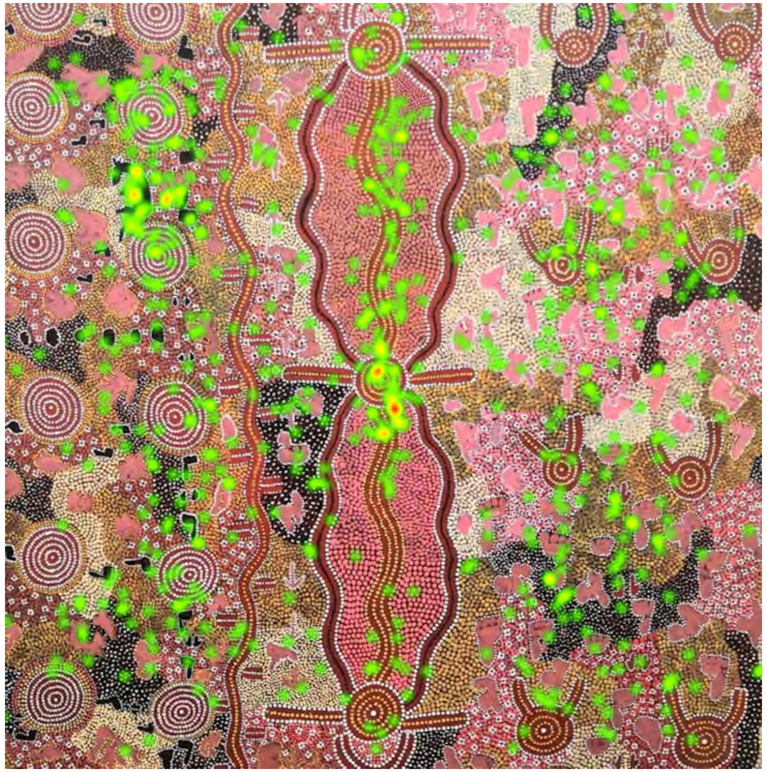

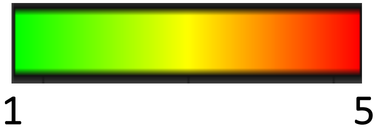

IA-9 - Total Fixation Count

Museum

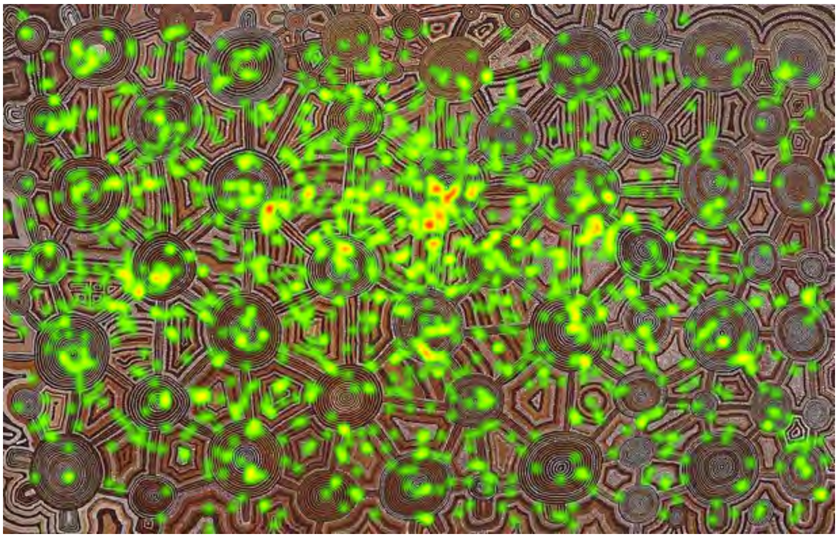

Screen – Full size

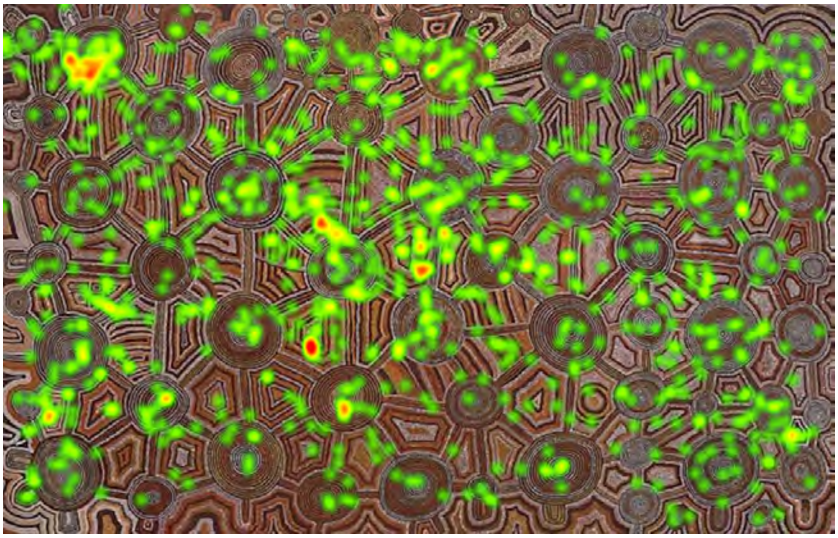

Screen – Relative Size

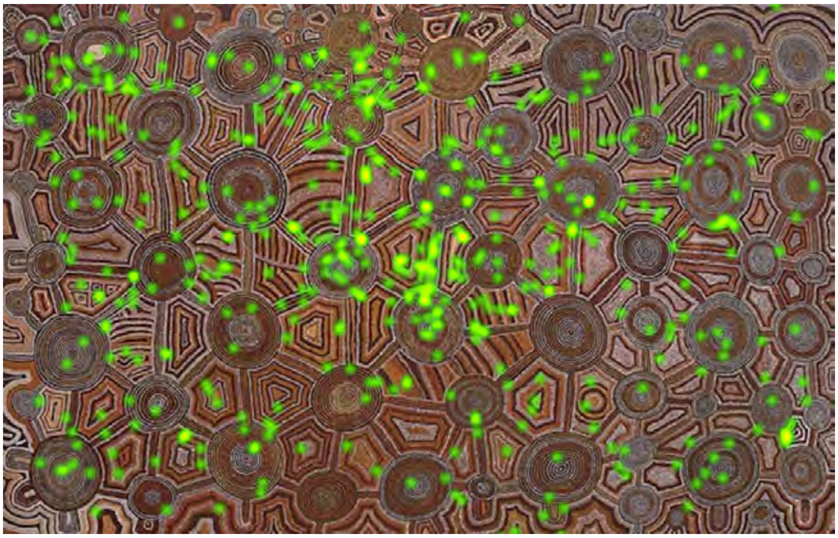

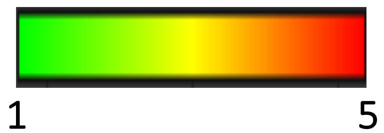

Supplement: Supplementary file 1 [file Data_Sheet_1.pdf]
